# The Influence of Chemical Activity Models on the Description of Ion Transport through Micro-Structured Cementitious Materials

**DOI:** 10.3390/ma16031116

**Published:** 2023-01-28

**Authors:** Krzysztof Szyszkiewicz-Warzecha, Grażyna Wilczek-Vera, Andrzej Lewenstam, Anna Górska, Jacek Tarasiuk, Robert Filipek

**Affiliations:** 1 Faculty of Materials Science and Ceramics, AGH University of Science and Technology, Al. Mickiewicza 30, 30-059 Kraków, Poland; 2 Department of Chemistry, McGill University, 845 Sherbrooke Street West Montreal, Montreal, QC H3A 0G4, Canada

**Keywords:** multi-ion transport modeling, Nernst–Planck–Poisson equations, cementitious materials, activity of ions, concentrated electrolyte, Pitzer model, X-ray computed tomography, 3D concrete microstructure

## Abstract

The significance of ion activity in transport through a porous concrete material sample with steel rebar in its center and bathing solution is presented. For the first time, different conventions and models of ion activity are compared in their significance and influence on the ion fluxes. The study closes an interpretational gap between ion activity in a stand-alone (stagnant) electrolyte solution and ion transport (dynamic) through concrete pores. Ionic activity models developed in stationary systems, namely, the Debye–Hückel (DH), extended DH, Davies, Truesdell–Jones, and Pitzer models, were used for modeling the transport of ions driven through the activity gradient. The activities of ions are incorporated into a frame of the Nernst–Planck–Poisson (NPP) equations. Calculations were done with COMSOL software for a real concrete microstructure determined by X-ray computed tomography. The concentration profiles of four ions (Na^+^, Cl^−^, K^+^, OH^−^), the ionic strength, and the electric potential in mortar (with pores) and concrete samples (with aggregates and pores) are presented and compared. The Pitzer equation gave the most reliable results for all systems studied. The difference between the concentration profiles calculated with this equation and with the assumption of the ideality of the solution is negligible while the potential profiles are clearly distinguishable.

## 1. Introduction

The transport of ions in liquid-filled pores in cementitious materials is one of the limiting factors that determine the durability of cement, in particular through corrosion of the reinforcement in concrete structures. Thus, understanding and providing a quantitative description of ion transport in cementitious materials is crucial to limit/minimize reinforcement corrosion in concrete and hence is beneficial for the environment and an important element of sustainable development [1,2].

### 1.1. Modeling Ion Activity

An ion’s charge and chemical properties determine its activity in liquid and solid media. Consequently, the electrostatic and electrodynamic behavior of ions depends on the ionic activity. Ionic activity determines a thermodynamic driving force of all electrochemical processes, and has, therefore, been the focus of modern chemistry since the beginning of the 20th century [3,4,5]. Starting with the fundamental works of Debye, Hückel, and Onsager, multiple approaches have been proposed to develop a theory of ion activity and ion activity coefficients that relate ion activity to ion concentration. Direct interpretations of activity coefficients in different matrices and mixtures have been discussed by different authors [6,7,8,9,10,11,12,13,14,15,16,17,18]. Typically, the outcomes converge for low concentrations of a single strong binary electrolyte of monovalent ions, such as KCl.

However, for higher ionic concentrations, unsymmetrical electrolytes, and multi-ionic systems, the outcomes do not converge. The reasons are multiple. Founding a unified convention that expresses ionic nonideality in high concentrations and increased anisotropy of the matrices is the most challenging. Obviously, the behavior of ions in close-to-saturation pore-filling solutions represents a top-challenge, which makes direct ionic activity predictions diverse and case-related.

### 1.2. Modeling Ion Transport in Cementitious Materials

The topic of ion transport in cement-based media has been extensively explored by scientists over the last few decades, e.g., [19,20,21,22,23,24,25,26,27,28,29,30,31,32,33,34,35,36,37,38,39,40]. Most papers [19,20,23,24,27,32] consider one-dimensional models, and many models neglect the influence of ion activities on transport [19,20,23,24,25,27,28,29,30,33]. Many papers concentrate on chloride ions diffusion only, neglecting other ions and their interactions [19,20,25,27,29,30]. A limited number of papers include the activity gradient as a driving force [16,21,22,34,36], at least in the form of the Debye–Hückel model of activity coefficients.

In time, researchers realized that cement microstructure should be incorporated while modeling transport in cementitious materials [38,39,40], however 3D models including cement real microstructure are still rare and models describing ion transport are very simplified, e.g., simple diffusion [41], although this topic is extensively covered in the field of battery modeling [42,43,44,45,46,47,48].

There is a lack of papers where both ion activities are taken into account and the 3D cementitious material microstructure is investigated. This paper describes the method of an indirect assessment of ionic activity, which takes advantage of the influence of ion activity on ion transport in porous concrete materials via numerical simulations of ion fluxes. Owing to the numerical access of the magnitude of ion fluxes, including the electrical potential effects, a basis for an indirect assessment of ion activity is developed. This approach provides a unique tool to help elucidate or even evaluate the validity of different approaches directly predicting ion activities.

## 2. Model of Multi-Ionic Transport in Cementitious Materials

Mass transfer in electrolytes is based on a material balance equation (mass conservation), description of species movement (constitutive relation for fluxes), identification of possible reactions (homogeneous (in bulk) and heterogeneous (at interfaces)), Poisson’s equation for electric potential, and models for computing activity coefficients of ions. Moreover, we also must account for the porous nature of concrete materials. Descriptions of ion transport in electrolyte solutions and porous materials are presented in Appendix A. Relevant equations will be presented in the following sections.

### 2.1. Chemical Composition of the System Studied

Numerous studies on the properties of the liquid phase in cementitious materials reveal that in the pores of cement paste at a water-to-cement ratio of 0.5–0.6, the following ions are present Na^+^, K^+^, Ca^2+^, SO_4_^2−^, and OH^−^ [49]. Concentrations of Na^+^, K^+^, and OH^−^ ions are high, so the liquid phase is largely the relatively concentrated solution of sodium and potassium hydroxide. Taylor [50] and Longuet et al. [51] report that the typical concentrations in the liquid phase of cement paste are as follows: Na^+^: 0.05–0.2 mol dm^−3^ and K^+^: 0.2–0.5 mol dm^−3^, at pH 13.4–13.8, respectively. The ions considered in our system are the free ions of Na^+^, K^+^, Cl^−^, and OH^−^, which means that side reactions such as ionic complexation or adsorption are excluded. In this study, we also neglect the presence of bivalent ions and carbon dioxide.

### 2.2. Transport of Ions in Cementitious Materials

In our calculations, we consider a 2D model of reinforced concrete in which ions move. This causes different concentration distributions and passage of local electric current, as well as different electric potential distributions. Four ions present: Na^+^, Cl^−^, K^+^, and OH^−^ are denoted by integers 1, 2, 3, and 4 and have charge numbers $z_{1}=+1,\;z_{2}=-1,$$\;z_{3}=+1,\;z_{4}=-1$.

**Figure 1:**
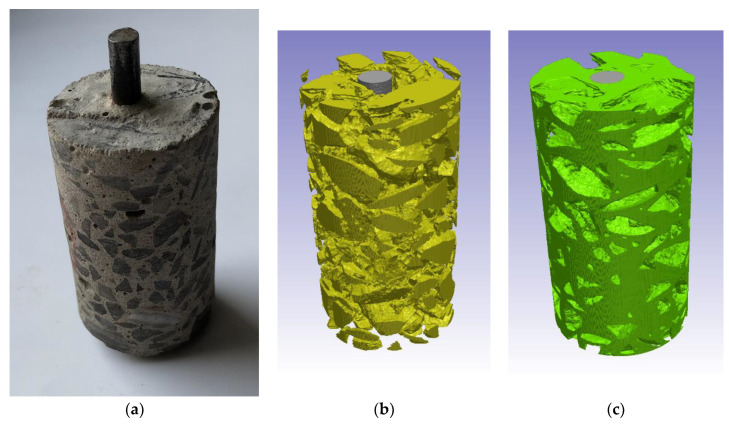
A photograph of the concrete sample for which simulations were performed (**a**). Computed tomography reconstruction of: (**b**) aggregates in the sample—yellow color, and (**c**) mortar—green color. The steel rod is shown in gray.

In this paper, we consider a process of multi-component transport occurring inside the concrete cover of a reinforcing bar (rebar). The setup is a cylindrical rebar with a concrete coating submerged in an external electrolyte that fills a cubic container, as shown in Figure 1a. The concrete cover is a porous medium (a network of connected micropores). The electrolyte can penetrate the pore system from the external electrolyte.

The simulations were performed for samples of a cylindrical shape, 25 mm in diameter and 50 mm in height, with an embedded steel rod. Figure 1a presents a photograph of the concrete sample for which simulations were performed. The 3D microstructure of the concrete sample was obtained by X-Ray Computed Tomography—for further details see Appendix B. The detailed positions of the mortar, aggregates, and steel rebar were identified by a segmentation procedure performed by the SimpleWare ScanIP software [52] (Figure 1b,c).

**Governing equations**

The whole simulation region is divided into four subregions (electrolyte, liquid in pores of concrete, aggregates, and rebar). In the present model, aggregates and the rebar are not penetrable, i.e., they are effectively excluded from computations and their sole role is to provide the geometrical boundaries for two other regions: the external electrolyte and the pore system, where the governing equations are solved.

*External Electrolyte:*

Using the Nernst–Planck flux with activity coefficients corrections, mass balance law, Poisson’s equation for electric potential, and effective diffusion coefficient to account for porous concrete matrix, the following set of partial differential equations (PDEs) inside the electrolyte is obtained

(1) $$\left\{\begin{array}{l} \frac{\partial c_{i}}{\partial t}\;+\;\nabla \cdot J_{i}=\;\;\;0,\;\;\;\;\;\;J_{i}\;=-D_{i}\nabla c_{i}\;-\;\;z_{i}D_{i}\frac{F}{RT}c_{i}\nabla \varphi \;\;+\;\;D_{i}c_{i}\nabla (\ln\gamma_{i}),\;\;\;\;\;\;i=1\ldots 4,\\ \nabla \cdot (-\varepsilon_{0}\varepsilon_{r}\nabla \varphi )=F\cdot \sum_{i=1}^{4}z_{i}c_{i}. \end{array}\right.$$

*Pore system:*

Using the homogenization technique [53,54], the Nernst–Planck flux with activity coefficients corrections, mass balance law, and Poisson’s equation for electric potential, gives the following set of PDEs inside the pore electrolyte:

(2) $$\left\{\begin{array}{l} \phi \frac{\partial c_{i}}{\partial t}\;+\;\nabla \cdot J_{i}=\;\;\;0,\;\;\;\;J_{i}\;=-D_{i}^{eff}\nabla c_{i}\;-\;\;z_{i}D_{i}^{eff}\frac{F}{RT}c_{i}\nabla \varphi \;\;+\;\;D_{i}^{eff}c_{i}\nabla (\ln\gamma_{i}),\;\;\;i=1\ldots 4,\\ \nabla \cdot (-\varepsilon_{0}\varepsilon_{r}\phi \;\;\tau \;\nabla \varphi )=F\cdot \phi \sum_{i=1}^{4}z_{i}c_{i}, \end{array}\right.$$

where ϕ is the porosity, *τ* is the tortuosity and $D_{i}^{eff}$ is the effective diffusion coefficient of *i*-th ion in the hydrated cement phase.

**Boundary conditions**

*Ions:*

In the first approach, we assume that the system is closed, i.e., the external boundary of a container containing a concrete sample immersed in an external solution is impenetrable for ions:

(3) $$-n\cdot J_{i}=0,\;\;\;\;i=1,2,3,4,$$

where n is the normal vector on the boundary. The same condition is also used on the boundary of the rebar (no anodic and cathodic reactions).

At the junction between the electrolyte phase and the concrete coating, there is no true boundary condition and, in computations, we ensure that the flux is continuous.

*Electric potential:*

The container is assumed to be of stainless steel, so the electric potential is constant on the top part and because there are not redox reactions on its surface, we can also assume that the normal component of the potential gradient is zero:

(4) $$\begin{array}{ll} \varphi =0 & \mathrm{on}\;\mathrm{the}\;\mathrm{top}\;\mathrm{side}\;,\\ -n\cdot (\varepsilon_{0}\varepsilon_{r}\nabla \varphi )=0 & \mathrm{on}\;\mathrm{the}\;\mathrm{left},\;\mathrm{right},\;\mathrm{and}\;\mathrm{bottom}\;\mathrm{part}. \end{array}$$

**Initial conditions**

*Ions:*

At the beginning of the process, an aqueous solution of potassium hydroxide (case 1) and potassium and sodium hydroxide solution (case 2) was set inside the pores of the concrete as shown in Table 1. The external solution consists of an aqueous solution of sodium chloride of various concentrations, presented in Table 2. Hence, the initial concentration of ions in the liquid in pores and the external solution are:

(5) $$\begin{array}{ll} c_{i}(x,0)=c_{i,ext}^{0} & \mathrm{for}\;\mathrm{external}\;\mathrm{solution},\\ c_{i}(x,0)=c_{i,pore}^{0} & \mathrm{for}\;\mathrm{liquid}\;\mathrm{in}\;\mathrm{pores}. \end{array}$$

*Electric potential:*

Initial ion concentrations satisfy the electroneutrality condition and consequently, we assume that the initial potential in the concrete sample and external solution is zero.

## 3. Chemical Activity of an Individual Ion and Mean Ionic Activity Coefficient

When an electrolyte, $C_{v_{+}}A_{v_{-}},$ dissolves in water, it dissociates, partially or almost completely, into $v_{+}$ cations (charge number z_+_) and $v_{-}$ anions (charge number z_—_):

(6) $$C_{\nu_{+}}A_{\nu_{-}}⇌v_{+}\;C^{\;z_{+}}+\;\;v_{-}\;A^{z_{-}}$$

The extent of this dissociation is described by its equilibrium constant, $K_{eq}(T)$, expressed in terms of activities of the cation, $a_{+}$, the anion, $a_{-}$, and the electrolyte, $a_{E}$

(7) $$K_{eq}(T)=\frac{a_{+}^{v_{+}}a_{-}^{v_{-}}}{a_{E}}.$$

The activities, $a_{i},$ are dimensionless “*corrected concentrations*” of species that take into account complicated ion–ion or ion–solvent interactions. These corrections are accounted for by the single ion activity coefficients (SIACs), $\gamma_{i}\;$.

When units of molality are used to express the concentration of the electrolyte, the single ion activity is given by

(8) $$a_{i}={\tilde{m}}_{i}\;\;\;\gamma_{i}=v_{i}\;\;\tilde{m}\;\;\;\gamma_{i},$$

where ${\tilde{m}}_{i}$ is the dimensionless molality of the ion *i* and $\tilde{m}$ is the dimensionless molality of the electrolyte solute. The dimensionless molality is the molality divided by 1 [mol/kg of solvent]. In more general terms, a dimensionless concentration is a concentration divided by 1 with the same units of concentration. Note, that the values of the activity do not depend on the units of concentration, but the values of the activity coefficients, although dimensionless, do, and they need to be consistent with the units of concentration used.

At equilibrium,

(9) $$a_{E}=a_{+}^{v_{+}}a_{-}^{v_{-}}={\left({\tilde{m}}_{+}\;\gamma_{+}\right)}^{v_{+}}{\left({\tilde{m}}_{-}\;\gamma_{-}\right)}^{v_{-}}={\tilde{m}}^{v}\gamma_{\pm }^{v}(v_{+}^{v_{+}}v_{-}^{v_{-}}).$$

Here, $\gamma_{+},\;\;\;\gamma_{-}$ are the single ion activity coefficients (SIACs) of the cation and anion, respectively. The mean ionic activity coefficient of the electrolyte (MIAC), $\gamma_{\pm }$, is defined as

(10) $$\gamma_{\pm }^{v}=\gamma_{+}^{v_{+}}\;\gamma_{-}^{v_{-}}\;\mathrm{with}\;v=v_{+}+v_{-}.$$

At infinite dilution, all activity coefficients of ions, MIACs, and SIACs, tend to unity.

For detailed coverage of the thermodynamics of aqueous electrolyte solutions see [6,7].

Note that the MIACs are well defined and the experimental values for many electrolytes in aqueous or nonaqueous solutions can be found in various sources, for example [9,10]. However, the SIACs are, using the expression of Bates [11] “*elusive*” properties. Guggenheim [12] called them “*a quantity which physically does not exist*”. Nevertheless, according to Pitzer, ”*for many practical applications to complex mixtures, it is simpler to use single-ion expressions*”([7], p. 298). A recent review of the topic of SIACs can be found in [7].

## 4. Chemical Activity Models

The work on the development of a theory of electrolytes started at the beginning of the 20th century and is still a work in progress. The interested reader will find a brief description of the most used models in [13]. Table 3 summarizes the most popular models used, together with the ranges of their applications.

For reader convenience, concise descriptions of the above-mentioned activity models are presented in Appendix C. Models listed in Table 3 were developed based on the experimental values of the mean ionic activity coefficients with the occasional addition of the experimental values of the osmotic pressure and other properties of electrolyte mixtures. Based on these, mostly practical, formulas, the equations for the individual activity coefficients were then deduced with the use of assumptions discussed in Appendix C.

The main goal of the paper is to assess the impact of ionic activities on the dynamic behavior of concentrations, current, and potential distribution in concrete. For comparison the most successful models of activity coefficients have been selected: the *Extended Truesdell–Jones* model, the model based on the *Pitzer theory* of electrolytes, and the *Pitzer model using MacInnes scaling*. Below we present a short description and relevant formulas.

### 4.1. Truesdell–Jones Model and Extended Davies Models

The Truesdell–Jones equation used in the paper has the form:

(11) $$\log\;\;\gamma_{i}\;=-\frac{Az_{i}^{2}\sqrt{I}}{1+a_{i}B\sqrt{I}}\;\;+\;\;b_{i}I.$$

For water solutions at 1 bar pressure at 25 °C A = 0.5094 mol^−1/2^·dm^1/2^ and B = 0.3289 mol^−1/2^·dm^1/2^·Å^−1^, *I* is in molarity units (mol·dm^3^). The parameters $a_{i}$ and $b_{i}$ are listed in Table 4. As the value of the ionic radius is unusually high at 10.65 Å, we also performed calculations with a more common value of 3.5 Å and compare the results (see Section 6). *Note*: the units of parameters and units of ionic strength must be consistent with each other.

In [16], Samson et al. approximated the more complex Pitzer model with a modified Truesdell–Jones equation, which considers a linear decrease in the coefficient C in Equation (A14) from its initial value of 0.2 [16]:

(12) $$\log\gamma_{i}=-\frac{Az_{i}^{2}\sqrt{I}}{1+a_{i}B\sqrt{I}}\;\;+\;\;\frac{(0.2-4.17\cdot 10^{-5}I)Az_{i}^{2}I}{\sqrt{1000}}.$$

This fit was performed in the range of concentrations up to 1500 mM.

The parameters *A* and *B* are defined as before and the ionic strength *I* is expressed in the mM units, (mmol dm^−3^ or mol m^−3^). Samson et al. [16] called Equation (12) the “*Extended Davies Model*”. The modeling was performed with all values of $a_{i}$ = 3 Å. Subsequently, the authors “fine-tuned” their model to Pitzer’s curves by adjusting the individual values of the effective radii for two ions: Cl^−^ and K^+^ (see the lower row in Table 5).

The parameters in Equation (12) must have appropriate dimensions to assure the dimensionless of the whole expression.

### 4.2. Pitzer Model

The Pitzer model is a generalization and improvement of the Guggenheim equations [17,18] for activities. Equations for osmotic coefficients were developed as well, but they are not pertinent to this paper. The Guggenheim equations are the basis of the Specific Ion Interaction Theory (SIT) covered in more detail in Appendix C.

(13) $$\ln\gamma_{MX}=-\frac{A_{\gamma }|z_{M}z_{X}|\sqrt{I}}{1+\sqrt{I}}\;\;+\;\;\frac{2\nu_{+}}{\nu_{+}+\nu_{-}}\sum_{a}\beta_{M,a}m_{a}\;\;+\;\;\frac{2\nu_{-}}{\nu_{+}+\nu_{-}}\sum_{c}\beta_{c,X}m_{c},$$

where the sums are over all anions $\left(\sum_{a}\right)$ and cations $(\sum_{c}),$ respectively, and $A_{\gamma }$ is the usual Debye–Hückel coefficient. The quantities *β* are constants (at given *T*) that represent the net effect of various short-range interactions between ions *M* and *X*. Terms pertinent to like-charged ions are excluded.

Using Guggenheim’s approach as a starting point, Pitzer developed a thermodynamically sound theory of ionic activities in electrolytes and obtained the following expressions for the activity coefficients of cation (*M*) and anion (*X*) in an aqueous solution of electrolyte MX. *Note*: there are no neutral solutes present, *c* denotes cations, and *a* denotes anions.

(14) $$\begin{array}{l} \ln\gamma_{M}=z_{M}^{2}F+\sum_{a=1}^{N_{a}}m_{a}(2B_{M,a}+ZC_{M,a})+\;\sum_{c=1}^{N_{c}}m_{c}(2\Phi_{M,c}+\sum_{a=1}^{N_{a}}m_{a}\psi_{M,ca})+\\ \sum_{a=1}^{N_{a}-1}\sum_{a^{\prime }=a+1}^{N_{a}}m_{a}m_{a^{\prime }}\psi_{aa^{\prime },M}+z_{M}\sum_{c=1}^{N_{c}}\sum_{a=1}^{N_{a}}m_{c}m_{a}C_{ca}, \end{array}$$

(15) $$\begin{array}{l} \ln\gamma_{X}=z_{X}^{2}F+\sum_{c=1}^{N_{c}}m_{c}(2B_{c,X}+ZC_{c,X})+\;\sum_{a=1}^{N_{a}}m_{a}(2\Phi_{X,a}+\sum_{c=1}^{N_{c}}m_{c}\psi_{X,a,c})+\\ \sum_{c=1}^{N_{c}-1}\sum_{c^{\prime }=c+1}^{N_{a}}m_{c}m_{c^{\prime }}\psi_{cc^{\prime },X}+|z_{X}|\sum_{c=1}^{N_{c}}\sum_{a=1}^{N_{a}}m_{c}m_{a}C_{ca}, \end{array}$$

where

(16) $$\begin{array}{l} F=-A^{\phi }\left[\frac{\sqrt{I}}{1+b\sqrt{I}}+\frac{2}{b}\ln(1+b\sqrt{I})\right]+\;\sum_{c=1}^{N_{c}}\sum_{a=1}^{N_{a}}m_{c}m_{a}{B^{\prime }}_{ca}+\\ \sum_{c=1}^{N_{c}-1}\sum_{c^{\prime }=c+1}^{Nc}m_{c}m_{c^{\prime }}{\Phi^{\prime }}_{cc^{\prime }}+\;\sum_{a=1}^{N_{a}-1}\sum_{a^{\prime }=a+1}^{N_{a}}m_{a}m_{a^{\prime }}{\Phi^{\prime }}_{aa}, \end{array}$$

(17) $$Z=\sum_{i}m_{i}|z_{i}|.$$

A concise summary of the equations and corresponding parameters can be found in [55]. The Pitzer Equations (14)–(17) for the system considered in our paper (NaCl-KOH in water) and all necessary parameters used in calculations are included in Appendix D.

It follows from the above equations that the single ion activity coefficients for 1:1 electrolytes have the same values for the cation, the anion, and the electrolyte itself.

### 4.3. Pitzer Model Using MacInnes Convention

In 1919, MacInnes [56] observed that for dilute (<0.1 M) aqueous solutions of chlorides of the alkali metals and hydrogen, “*the equivalent conductance of the chloride-ion constituent is, at any given concentration up to 0.1 N, substantially the same, whether the other ion-constituent associated with it is hydrogen ion or any of the alkali element ions*”. Therefore, he assumed that the activity of the chloride ion in these solutions is independent of the nature of the (univalent) positive ion and, in an aqueous solution of KCl, the K^+^ and Cl^−^ ions have equal activities.

(18) $$\gamma_{\mathrm{KCl}}=\gamma_{{\mathrm{Cl}}^{-}}=\gamma_{K^{+}}$$

With the help of mean ionic activity coefficients of chlorides, this assumption allows for the calculation of SIACs of univalent positive ions. For example, to calculate the SIAC of a sodium ion in sodium chloride solution, we get:

(19) $$\gamma_{{\mathrm{Na}}^{+}}=\frac{\gamma_{\mathrm{NaCl}}^{2}}{\gamma_{{\mathrm{Cl}}^{-}}}=\frac{\gamma_{\mathrm{NaCl}}^{2}}{\gamma_{\mathrm{KCl}}},$$

where the activity coefficient of the chloride ion in NaCl is equal to the activity coefficient of a KCl solution at the same ionic strength.

Analysing Pitzer equations, we see that for 1:1 electrolytes, they produce identical activity coefficients for univalent cations and anions, i.e.,

(20) $$\gamma_{+}=\gamma_{-}=\gamma_{\pm }.$$

The application of the MacInnes assumption permits the calculation of the activities of separate ions. Thus, to transform Pitzer’s activity coefficient for a species *i* to the MacInnes convention, it is necessary to multiply it by the ratio of the Pitzer activity coefficient of a chloride ion to the experimental value of the MIAC of a pure aqueous solution of KCl taken to the power of the charge of ion *i*. The expression in the bracket is calculated at the same ionic strength as the solution studied.

(21) $$\gamma_{i}^{MacInnes}=\gamma_{i}^{Pitzer}{\left(\frac{\gamma_{{\mathrm{Cl}}^{-}}^{Pitzer}}{\gamma_{\pm ,\;\;\mathrm{KCl}}}\right)}_{I}^{z_{i}}.$$

Equation (21) is normally used in a wide range of concentrations despite the inherent limitations of its applicability beyond the 0–0.1 M range. In addition, due to the limited solubility of KCl, the NBS (National Bureau of Standards) database provides experimental values of MIACs for KCl up to 5 M only. If the total ionic strength of the solution is higher than that, it is recommended to calculate the MIAC value for KCl from the Pitzer equation. In a solution that does not contain chloride ions, the bracket term in Equation (21) reduces to unity and the normalization is meaningless.

## 5. Numerical Calculations

### 5.1. Geometrical and Numerical Setup of Calculations

Calculations of activity models were implemented as a DLL library in Microsoft Visual Studio 2022 based on the expressions presented in Appendix D and calculations of transport of ionic specious in cementitious materials were implemented in the COMSOL environment using the user-defined General Form PDE Physics Interface. Activities of the ions were calculated as an external Dynamic-link Library (DLL) function evaluation. The solution was obtained using the Finite Element Method and the Lagrange shape function of quadratic element order and the Multifrontal Massively Parallel Sparse Direct solver (MUMPS).

**Figure 2:**
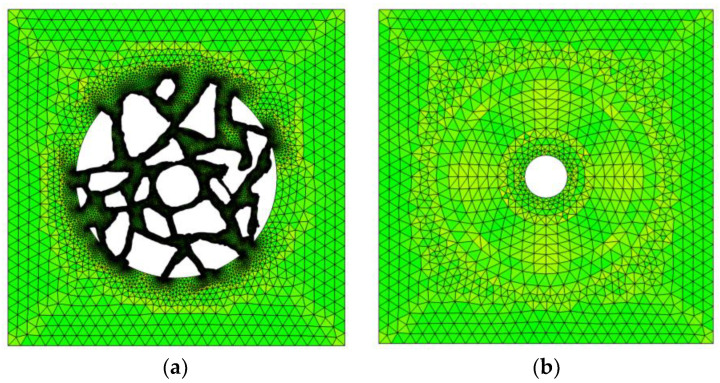
2D mesh used in numerical calculations of (**a**) cement and (**b**) mortar cylinder-shaped samples immersed in an aqueous solution.

Figure 2 shows the mesh used in the numerical calculations of (a) cement and (b) mortar samples. White areas in Figure 2a correspond to basalt aggregates, which do not contribute to ion transport.

The mesh for the concrete sample is built of more than 94,000 elements, while that of the mortar sample mesh is composed of over 2300 elements. The choice of the meshes was preceded by a detailed analysis of the problem solution dependence on the mesh size. The choice of mesh parameters is presented below using the example of the mesh for the concrete sample. The meshes tested are given in Table 6.

The average absolute and average relative errors for chloride ion concentrations and electric potential for different meshes from Table 6 are presented in Table 7.

For further calculations, the finer mesh was chosen—a compromise between satisfactory accuracy and moderate time of calculations.

### 5.2. Calculation of Density for Molality/Molarity Conversion

It is important to notice that in this paper, for all models of ion transport, the concentrations are defined as *molar concentrations*, while activity coefficients, depending on the model, are calculated in *molalities* (Pitzer model with or without MacInnes normalization) or molarities (Davies and extended Truesdell–Jones models). Thus, for example, when we apply the Pitzer model, the activity coefficients of ions are molal activities and they must be recalculated to molar activity coefficients using the Formulae (A31)—see Appendix E.

Knowledge of the density of a solution is necessary for conversions between molality and molarity concentrations. In the literature, there are available data for most binary and selected ternary systems, e.g., [57]. For an aqueous quaternary system consisting of the ions Na^+^, K^+^, OH^−^, or Cl^−^ no such data exist. The densities as a function of ionic molarities were calculated on the basis of the *solvate model of solutions* as proposed in [58].

Let us consider any binary salt dissolved in water. The density of a solution as a function of molarity *c* can be approximated by the formula

(22) $$d(c)=\;\;d_{0}+\;\;ac\;\;-\;\;\frac{bc^{2}}{d_{0}+ac},$$

where $d_{0}$ is the density of pure solvent (water at temperature 25 °C), and a,  b are parameters characteristic for a particular salt. If we deal with a mixture of several binary salts (including hydroxides) $M_{i}X_{i}$ for i=1,…,n, then the formula takes the following form

(23) $$d\;(c_{1},\ldots ,c_{n})\;\;=\;\;\;d_{0}\;\;\;+\;\;\sum_{i=1}^{n}a_{i}c_{i}-\;\;\;\frac{\sum_{i=1}^{n}M_{e,i}c_{i}}{d_{0}+\sum_{i=1}^{n}a_{i}c_{i}}\;\;\sum_{i=1}^{n}\frac{a_{i}c_{i}}{M_{e,i}},$$

where $M_{e,i}$ is the molecular weight of electrolyte $M_{i}X_{i}.$ Calculated parameters *a* and *b* for selected binary electrolytes are presented in Table 8.

More details of this model and comparison with results from other models calculating the density of an aqueous multi-ion electrolyte are in Appendix F.

We also need formulas that express binary electrolyte molarities in terms of individual ion molarities. This can be obtained by writing down material balances for all ions present and solving the resulting linear system of equations. However, it turns out that the system is singular (the determinant of the matrix is zero), but it has a rank equal to #(of ions) − 1 if and only if the electroneutrality condition is satisfied. As this condition is assumed in the paper, we can compute the binary electrolyte concentrations in terms of ion molarities and one free parameter. In the case of four ions Na^+^, K^+^, OH^−^, and Cl^−^ we have:

(24) $$\left\{\begin{array}{l} c_{\mathrm{NaCl}}=c_{\mathrm{Cl}}-c_{K}+t,\\ c_{\mathrm{NaOH}}=c_{\mathrm{Na}}-c_{\mathrm{Cl}}+c_{K}-t,\\ c_{\mathrm{KCl}}=c_{K}-t,\\ c_{\mathrm{KOH}}=t. \end{array}\right.$$

Here, the free parameter “*t*” was selected to be the KOH concentration.

## 6. Comparison of Different Models for the Prediction of Activity Coefficients in Aqueous Systems of NaOH, KOH, NaCl, or KCl

Before applying different models of activity coefficients to complex multicomponent systems in dynamic situations, it is reasonable to verify first how they perform in the simplest possible case of representing mean ionic activity coefficients (MIACs) in pure aqueous solutions.

As described before, we used the extended Davies and Truesdell–Jones models as well as the Pitzer model with and without MacInnes normalization and compared their predictions with the experiment values of MIACs for NaOH, KOH, NaCl, and KCl in their aqueous solutions.

Two sets of data for the extended Davies model were used:

(i) the same ion radii equal to 3 Å will be called “extended Davies 1”;
(ii) ion radii of ions equal: Na^+^ = 3 Å, OH^−^ = 3 Å, K^+^ = 3.3 Å, Cl^−^ = 2 Å will be called “extended Davies 2”.

For Truesdell–Jones model two variants were considered as well:

(i) OH^−^ radius = 10.65 Å, b = 0.21—denoted as “Truesdell–Jones 1”;
(ii) OH^−^ radius = 3.5 Å, b = 0.21—denoted as “Truesdell–Jones 2”.

### 6.1. Comparison of the MIACs Predictive Capability of Different Models

#### 6.1.1. Mean Ionic Activity Coefficients in Aqueous Solutions of Hydroxides: NaOH(*aq*) and KOH(*aq*)

**Figure 3:**
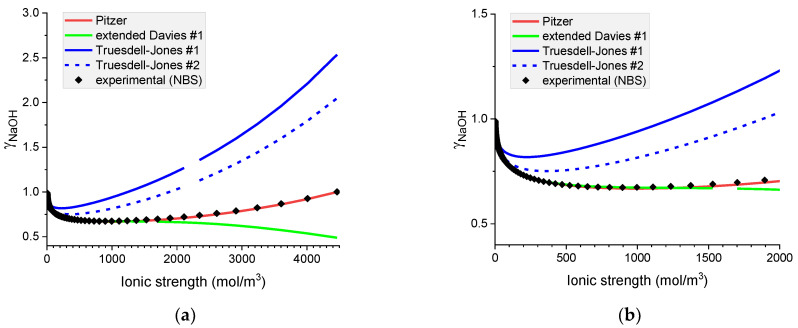
Molar mean ionic activity coefficients in aqueous NaOH for concentrated (**a**) and diluted (**b**) solutions. Comparison of calculated activity coefficients with experimental results (NBS) based on the Pitzer, extended Davies, and Truesdell–Jones models at 25 °C.

**Figure 4:**
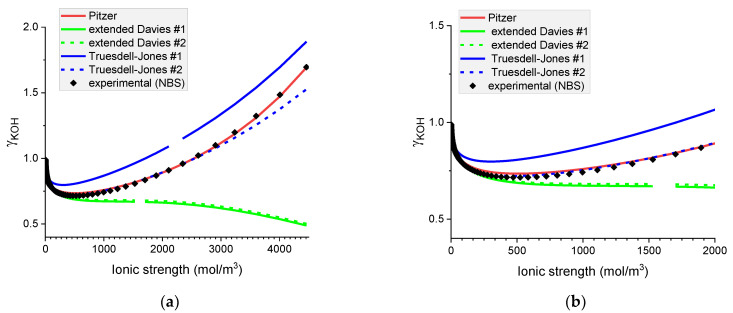
Molar mean ionic activity coefficients in aqueous KOH for (**a**) concentrated and (**b**) dilute solutions. Comparison of calculated activity coefficients based on the Pitzer, extended Davies, and Truesdell–Jones models with experimental results (NBS) at 25 °C.

Analysis of Figure 3 and Figure 4 shows that the Pitzer model represents well the mean ionic activity coefficients of sodium and potassium hydroxides in the whole range of concentrations. The extended Davies equation performs well for NaOH(*aq*) in ionic strengths up to 1200 mol m^−3^. This range narrows to around 500 mol m^−3^ for the solution of KOH(*aq*).

The predictive ability of the Truesdell–Jones equation in its original form is unexpectedly poor for both hydroxides.

#### 6.1.2. Mean Ionic Activity Coefficients in Aqueous Solutions of Chlorides: NaCl(*aq*) and KCl(*aq*)

**Figure 5:**
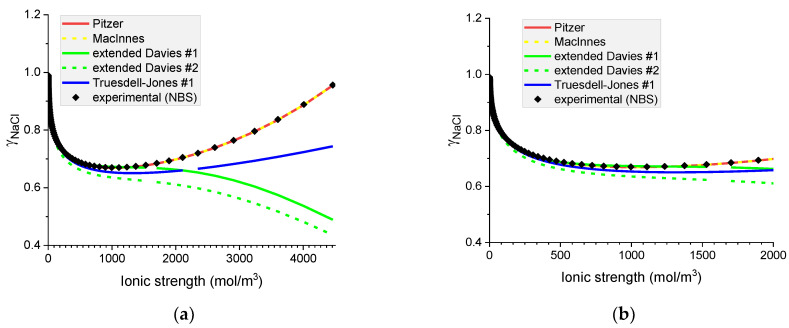
Molar mean ionic activity coefficients in aqueous NaCl for (**a**) concentrated, (**b**) dilute solutions. Comparison of calculated activity coefficients based on the Pitzer, MacInnes, extended Davies, and Truesdell–Jones models with experimental results (NBS) at 25 °C.

**Figure 6:**
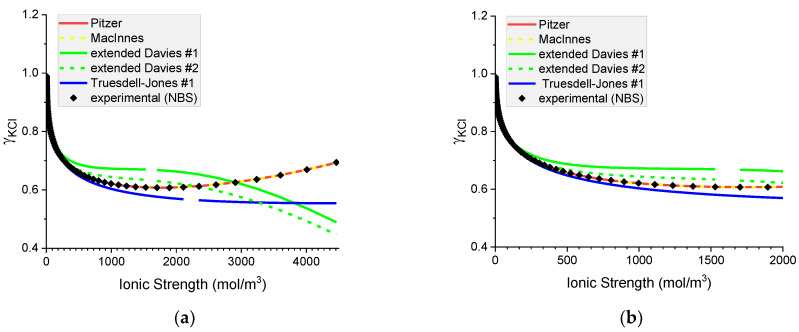
Molar mean ionic activity coefficients in aqueous KCl for (**a**) concentrated and (**b**) dilute solutions. Comparison of calculated activity coefficients based on the Pitzer, MacInnes, extended Davies, and Truesdell–Jones models with experimental results (NBS) at 25 °C.

In the aqueous chloride solutions of sodium and potassium, the mean ionic activity coefficients are very well predicted by the Pitzer model with and without MacInnes normalization (see Figure 5 and Figure 6). The extended Davies #1 equation predicts MIACc in NaCl solutions up to the concentration of 2000 mol m^−3^, but its performance is much worse in KCl solutions. Conversely, the extended Davies #2 equation with adjusted parameters performs worse for the NaCl solution than for the KCl solution, where it gives a good representation up to 2000 mol m^−3^. For concentrations above 2000 mol m^−3^, the extended Davies equation is not reliable. One should remember that Samson et al. [16] fitted the parameters of the equation up to around 1400 mol m^−3^ only. The Truesdell–Jones model works in NaCl solution up to 800 mol m^−3^ and in KCl solution up to 300 mol m^−3^ only.

As in the existing paradigm, only the experimental values of the mean ionic activity coefficients are known, the above comparison allows us to conclude that from all the models tested only the Pitzer equation gives consistently reliable predictions of MIACs in binary aqueous systems.

Note that for MIACs, the MacInnes normalization produces identical results to the unnormalized Pitzer equation.

### 6.2. Comparison of Individual Activity Coefficients in Binary Electrolytes

Notwithstanding the conclusion from Section 6.1, we still decided to compare the performance of all the models in calculating the individual activity of ions in the binary systems studied. We should remember, that by design, the best-performing model in Section 6.1, the Pitzer model, produces individual activity coefficients that in the 1:1 electrolyte systems are identical to each other and equal to the mean ionic activity coefficient of the electrolyte. The MacInnes normalization (see Equations (18) and (19)), cannot be used in the pure aqueous solution of hydroxides and it will produce identical individual activity coefficients for K^+^ and Cl^−^ in the KCl solution.

It is interesting to notice that the extended Davies and Truesdell–Jones models give identical results for the same ion, independently of the co-ion present. The results calculated with the extended Davies model versions #1 and #2 will differ only for K^+^ and Cl^−^ ions due to the modification of the model’s parameters.

However, only the Pitzer model has the ion–counter-ion interaction parameters that make the predictions of individual activity of ions different for different counterions present.

**Figure 7:**
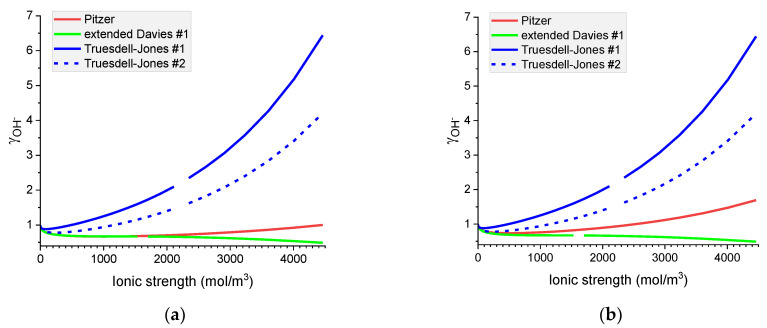
Molar OH^−^ individual activity coefficients in aqueous (**a**) NaOH and (**b**) KOH solutions. Comparison of calculated activity coefficients based on the Pitzer, extended Davies, and Truesdell–Jones models at 25 °C.

**Figure 8:**
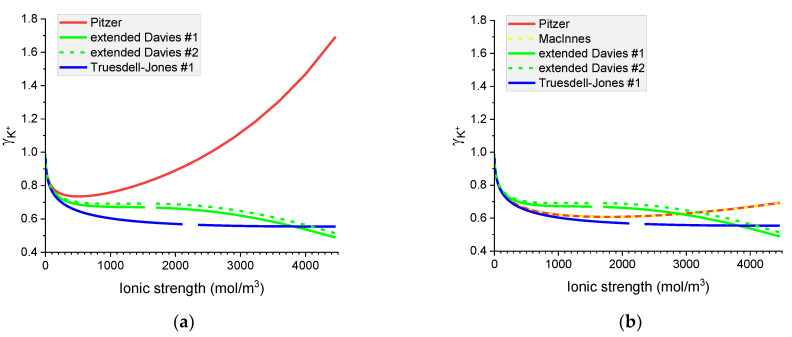
Molar K^+^ individual activity coefficients in aqueous (**a**) KOH and (**b**) KCl solutions. Comparison of calculated activity coefficients based on the Pitzer, MacInnes, extended Davies, and Truesdell–Jones models at 25 °C.

**Figure 9:**
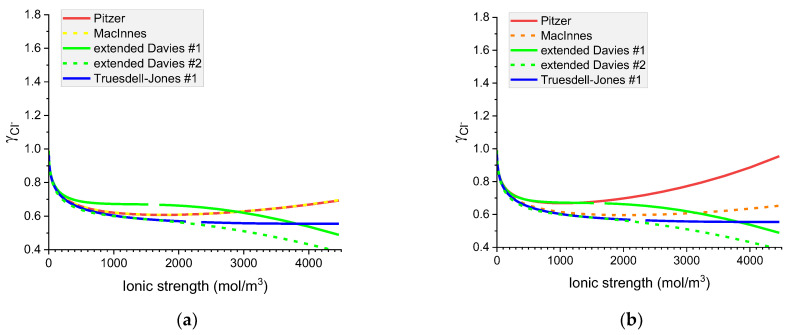
Molar Cl^−^ individual activity coefficients in aqueous (**a**) KCl and (**b**) NaCl solutions. Comparison of calculated activity coefficients based on the Pitzer, MacInnes, extended Davies, and Truesdell–Jones models at 25 °C.

**Figure 10:**
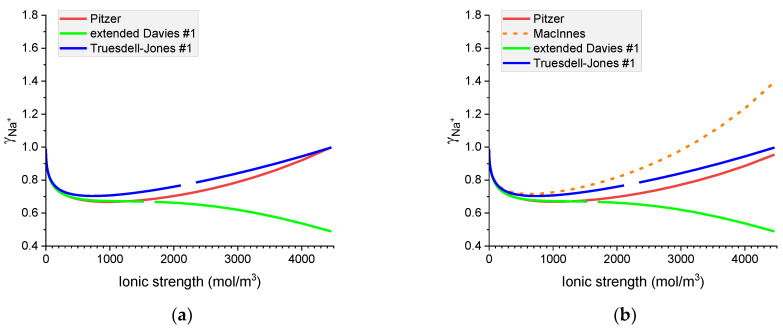
Molar Na^+^ individual activity coefficients in aqueous (**a**) NaOH and (**b**) NaCl solutions. Comparison of calculated activity coefficients based on the Pitzer, MacInnes, extended Davies, and Truesdell–Jones models at 25 °C.

Figure 7, Figure 8, Figure 9 and Figure 10 show that the calculated individual activity coefficients differ greatly for different models used and for various systems.

## 7. Individual Activity Coefficients for Quaternary K-Na-OH-Cl Water Electrolytes

In this section, we tested the sensitivity of the calculations due to the simplifying assumption of using in the calculations 590 mol m^−3^ KOH solution instead of the more realistic $120\;\mathrm{mol}\;m^{-3}$ NaOH and 470 mol m^−3^ KOH solution as an internal solution in the pre-conditioned concrete sample.

**Figure 11:**
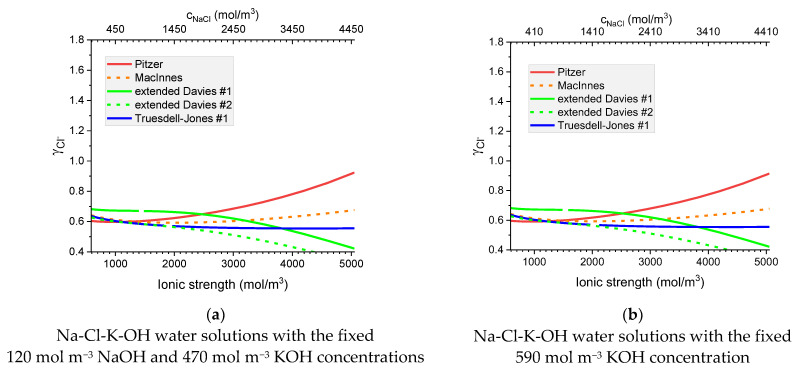
Molar individual activity coefficient of Cl^−^ in Na-Cl-K-OH water solutions as a function of ionic strength for the fixed: (**a**) KOH concentration 470 mol m^−3^; and NaOH concentration 120 mol/m^3^ and (**b**) KOH concentration 590 mol m^−3^. Notations #1 and #2 correspond to different ionic radii—see Table 4 and Table 5.

Thus, Figure 11 presents the calculated individual activity of ions in two solutions of the same concentration of OH^−^

(a) 120 mol m^−3^ NaOH and 470 mol m^−3^ KOH,
(b) 590 mol m^−3^ KOH,

as a function of the ionic strength when NaCl solution is added.

The analysis of the resulting graphs showed that the results of the calculations for cases (a) and (b) were visually virtually indistinguishable. As an example, we present in Figure 11 the case of chloride ions. Graphs for the rest of the ions are available in Supplementary Materials S1.

The effect of a different solution used for calculations is the most pronounced at the lowest ionic strength, but even there it is negligible. Thus, in the subsequent calculations, $590\;\mathrm{mol}\;m^{-3}$ KOH solution was used as an internal solution of the sample used for its pre-conditioning.

## 8. Chemical Activity Effects on Ionic Transport and Potential Distribution in Homogenous Cementitious Material (Mortar)

**Figure 12:**
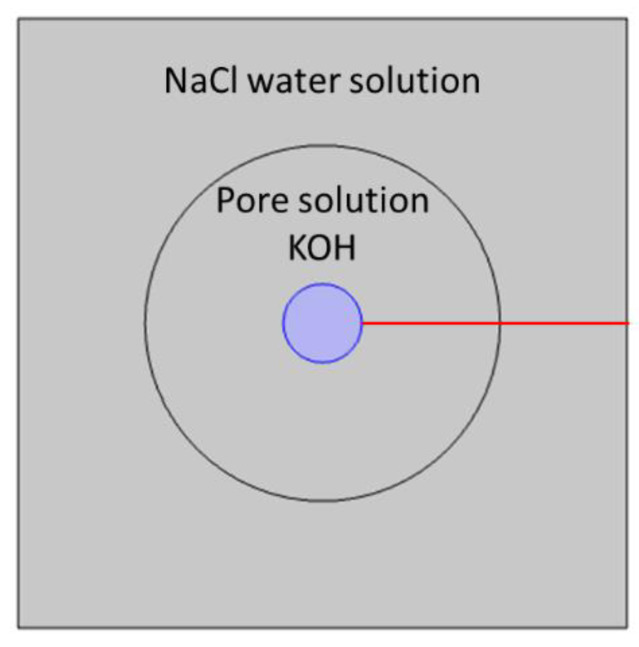
Model geometry—mortar sample of cylindrical geometry with an iron rod inside immersed in NaCl water solution. The results in Figure 13, Figure 14, Figure 15, Figure 16, Figure 17 and Figure 18 will be presented along the red line.

The influence of different activity models—Pitzer, MacInnes, extended Davies, and Truesdell–Jones—on ion transport for a model mortar cylindrical sample immersed in 20 wt% NaCl water solution is presented below. The mortar sample was modeled as an isotropic porous material with 16% porosity. In these calculations, for simplicity, we assumed that the pore solution is composed of 0.59 M KOH. We neglected the ions present in negligibly low concentrations (Na^+^, Ca^2+^, SO_4_^2−^). Calculations were performed in 2D cylindrical geometry. Calculated concentrations and electric potential profiles due to the system symmetry will be presented along the selected direction—the red line in Figure 12.

### 8.1. The Influence of Dielectric Permeability on Transport of Ions and Potential Distribution

It is known that the dielectric constant of a solution depends on its temperature and composition. Thus, in this part of our study, we investigate the sensitivity of calculated distributions of ion concentrations and the solution potential due to the dielectric constant changes.

**Figure 13:**
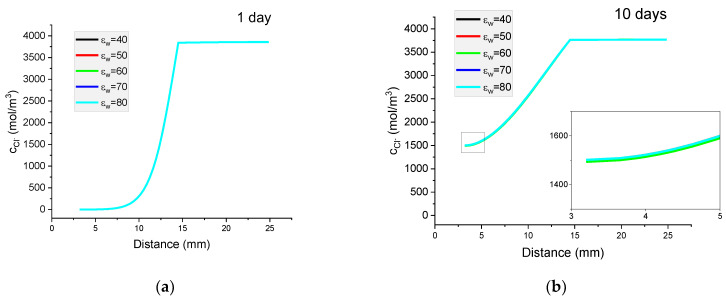

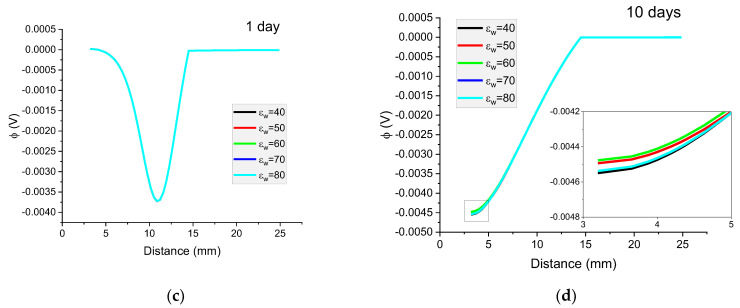
The influence of relative permeability on calculated concentration profiles of chloride ions in the mortar immersed in 20 wt% NaCl water solution after (**a**) 1 day; (**b**) 10 days; and potential distribution after (**c**) 1 day and (**d**) 10 days.

Figure 13 presents the results of the calculated concentration distributions for Cl^−^ ions as well the potential distributions of the solution after diffusion of 1 or 10 days. The value of the dielectric constant was varied from 40 to 80.

It can be seen that for the concentration profile of Cl^−^ ions, the effect of the change of the dielectric value within the range 40–80 is negligible and the maximum effect for the potential is of the order of 0.5 mV. For all other ions, this effect is negligible.

### 8.2. Ion Activities vs. Concentrations of Ions in Transport of Ions

**Figure 14:**
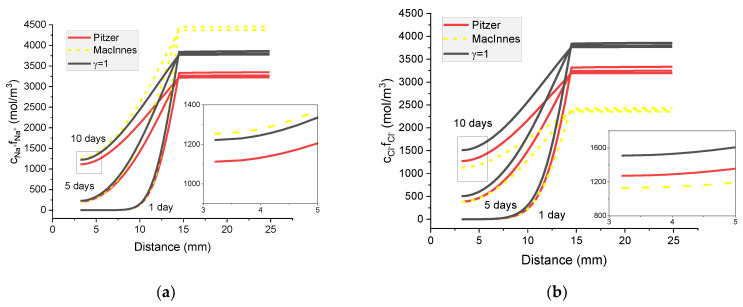

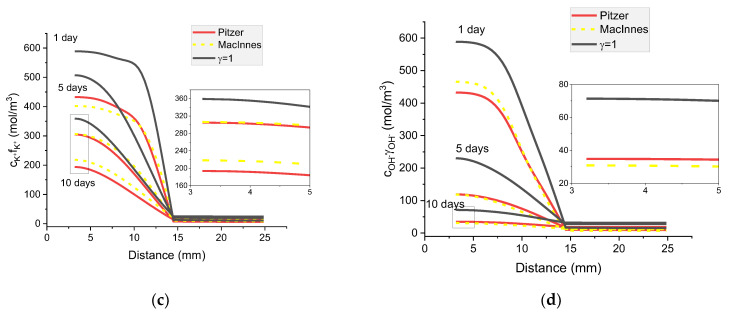
Calculated activity profiles of ions in the mortar immersed in 20 wt% NaCl water solution. (**a**) Na^+^; (**b**) Cl^−^; (**c**) K^+^; (**d**) OH^−^ for the Pitzer model and MacInnes scaling—comparison with ions concentrations (*γ* = 1).

**Figure 15:**
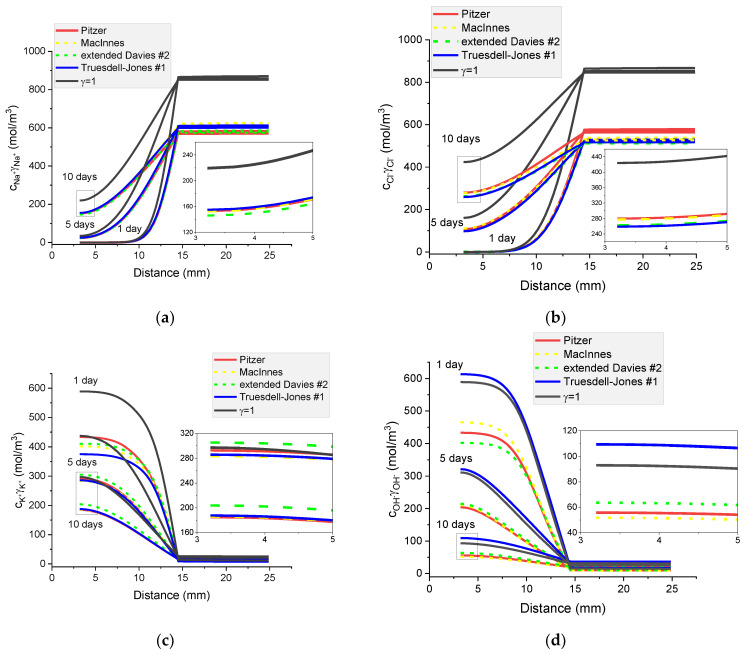
Calculated activities profiles of ions in the mortar immersed in 5 wt% NaCl water solution. (**a**) Na^+^; (**b**) Cl^−^; (**c**) K; (**d**) OH^−^ For the Pitzer model, MacInnes scaling, extended Davies and Truesdell–Jones models—comparison with ions concentrations (*γ* = 1).

Individual activities of ions calculated with the Pitzer model and using MacInnes normalization and ion concentrations for the concentrated external solution (20 wt% NaCl_(*aq*)_) are compared in Figure 14. In Figure 15, the calculated activities of ions for the Pitzer, MacInnes normalization, extended Davies, and Truesdell–Jones models are compared.

As expected, the effect of applying individual activity coefficients instead of treating the mixture of electrolytes as ideal is much more pronounced in the case of the concentrated external solution than in the dilute one. In the 20% wt. of aqueous NaOH, the activities of individual ions are substantially different from their concentrations, even after 10 days diffusion.

### 8.3. The Influence of Different Activity Coefficient Models on Concentration Profiles and Potential Distribution

**Figure 16:**
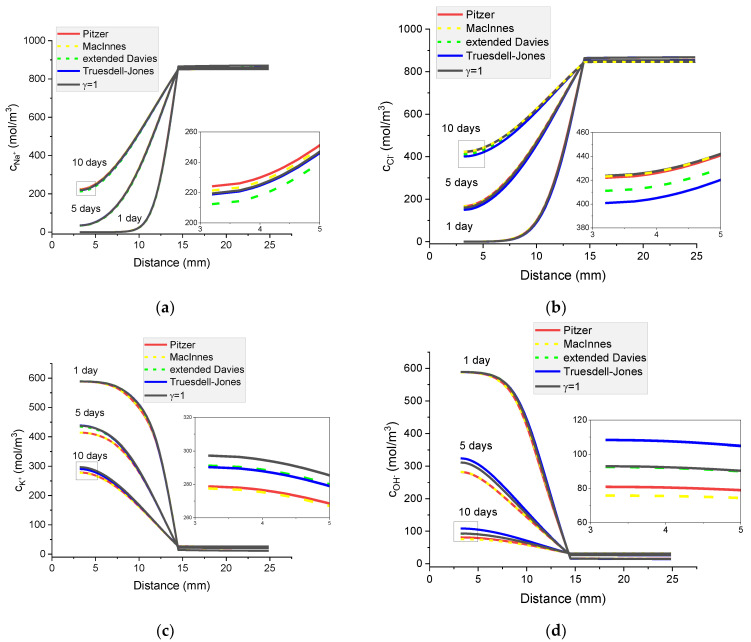

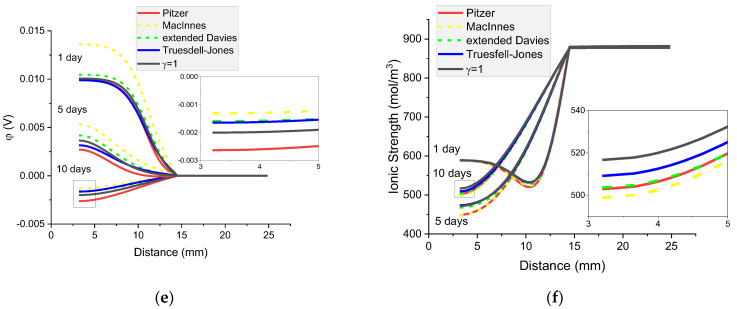
Calculated concentration profiles of ions in the mortar immersed in 5 wt% NaCl water solution. (**a**) Na^+^; (**b**) Cl^−^; (**c**) K^+^; (**d**) OH^−^; electrical potential (**e**) and ionic strength (**f**) after 1, 5, and 10 days.

Figure 16 shows the calculated Na^+^, Cl^−^, K^+^, and OH^−^ concentration profiles, electric potential distribution, and ionic strength distribution in a cementitious sample immersed in 5% NaCl water solution after 1, 5, and 10 days. After 1 day immersion, regardless of the activity coefficient model used, the concentration profiles do not differ from each other; moreover, they correspond very well to the case when the activity coefficient *γ* =1.

The influence of different activity models is clearly observed for electric potential profiles and these changes grow with time. Due to different ion diffusion coefficients and the fluxes interaction caused by the electric field, a non-monotonic ionic strength distribution with time is observed.

**Figure 17:**
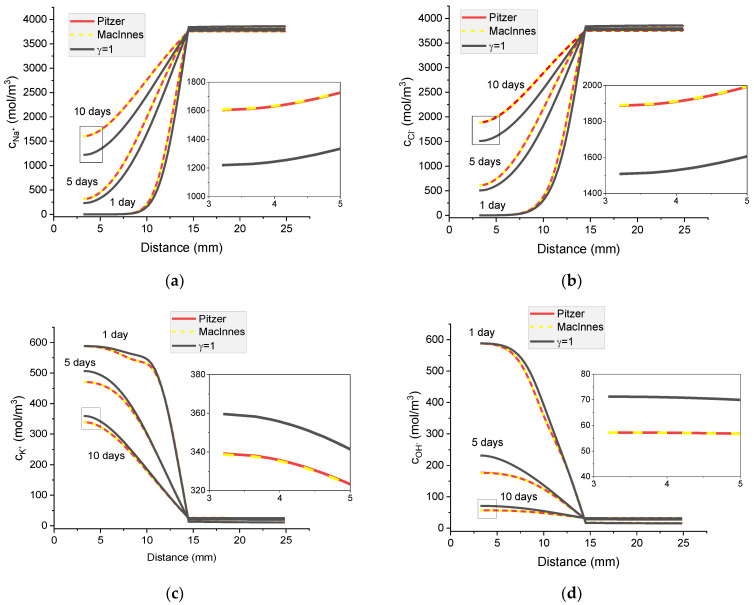

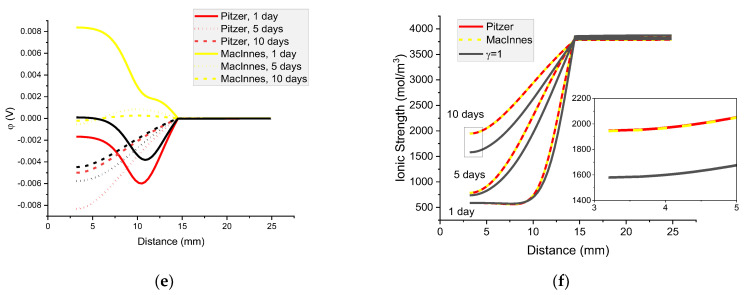
Calculated concentration profiles of ions in the mortar immersed in 20 wt% NaCl water solution. (**a**) Na^+^; (**b**) Cl^−^ Cl^−^; (**c**) K^+^; (**d**) OH^−^; electrical potential (**e**) and ionic strength (**f**). Pitzer model (red), MacInnes (yellow), ideal solution (γ = 1) (black) after 1, 5, and 10 days.

It can be seen in Figure 14 that the activities of individual ions are different when obtained using the Pitzer model and MacInnes normalization. While the calculated distributions of ion concentrations are the same for the Pitzer model and the MacInnes normalization, the potential distributions differ for both models (Figure 17). In the next section, the analysis of transport mechanisms for the Pitzer model and MacInnes normalization is carried out.

### 8.4. The Analysis of Transport Mechanisms and Its Influence on Potential Distribution

In the governing equations for the ionic flux $J_{i}$, we can distinguish separate terms due to the diffusion, migration, and activity of ion *i*:

(25) $$\begin{array}{l} \;\;\;\;\;\;\;J_{i}\;\;\;\;\;\;\;=\;\;\;\;-D_{i}\nabla c_{i}\;\;\;\;\;\;-\;\;\;\;\;\;z_{i}D_{i}\frac{F}{RT}c_{i}\nabla \varphi \;\;\;+\;\;\;D_{i}c_{i}\nabla (\ln\gamma_{i}).\\ \;\overset{}{\overset{︷}{total\;flux}}\;\;\;\;\;\overset{}{\overset{︷}{diffusion\;flux}}\;\;\;\;\;\;\;\;\overset{}{\overset{︷}{migration\;flux}}\;\;\;+\;\;\;\overset{}{\overset{︷}{activity\;flux}} \end{array}$$

**Figure 18:**
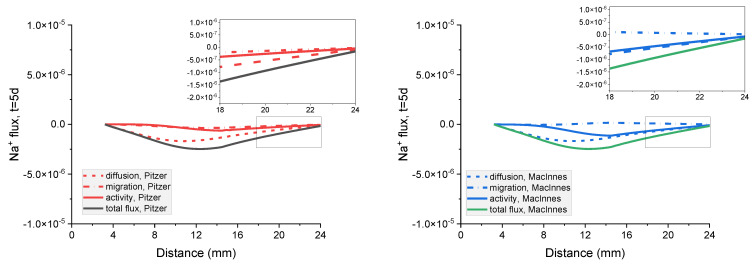

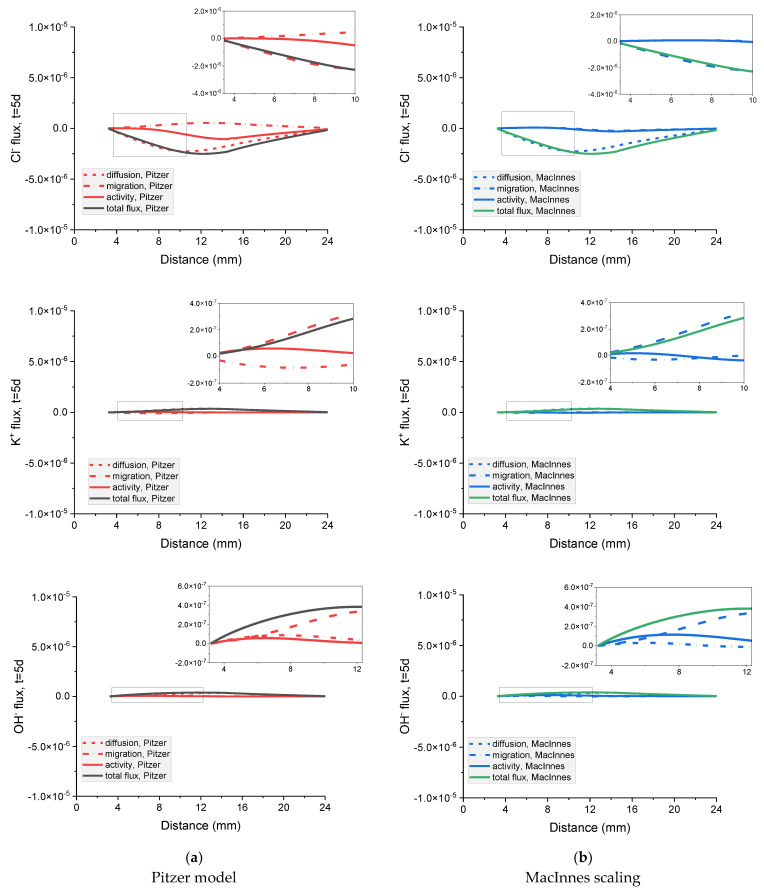
Influence of diffusion, migration, and activity terms on the total flux of Na^+^, Cl^−^ Cl^−^, K^+^, and OH^−^ ions in the mortar after 5 days immersion in 20 wt% NaCl water solution for (**a**) Pitzer and (**b**) MacInnes scaling.

Figure 18 shows the contribution of the particular flux terms to the calculated total flux for each ion in the system for a mortar sample immersed for 5 days in a concentrated (20%) external NaCl water solution. It is interesting to notice that the calculated diffusion flux is the same no matter if the MacInnes normalization was used or not in the Pitzer model. Conversely, the MacInnes normalization produces different results for the migration and activity fluxes, but their sum is the same as for the non-normalized Pitzer model.

The contribution of the particular flux terms to the calculated total flux for the mortar sample in dilute (5%) external NaCl water solution is presented in Supplementary Materials S2.

## 9. Chemical Activity Effects on Ionic Transport and Potential Distribution in Nonhomogeneous Cementitious Material (Concrete)

### 9.1. Concrete and Mortar Domains and Their Characterization

**Figure 19:**
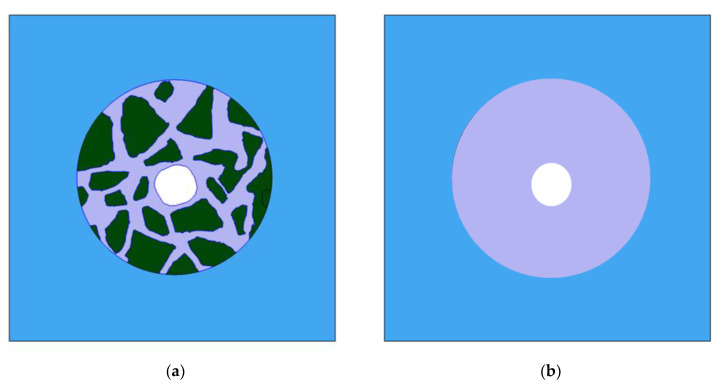
Simulation domains for concrete (**a**) and mortar (**b**) samples. Four regions can be identified: electrolyte (blue), liquid in pores of cement (dark grey), aggregates (dark green), and central rebar (white).

For tests, we selected a two-dimensional cross-section by a plane perpendicular to the axis of the central rebar (the white region in Figure 19) in two variants: (a) with aggregates (concrete) and (b) without aggregates (mortar).

Four ionic components are considered, which are characterized by charge numbers $z_{i}$, and diffusion coefficients of ions in electrolyte *D_i_* and liquid in the pores $D_{i}^{eff}$ (effective diffusivities)—see Table 9. The diffusion coefficients of ions in the external solution were assumed to be equal to their diffusion coefficients in an aqueous solution [36]. Effective diffusion coefficients of chloride ions were calculated using the inverse method and experimentally measured chloride penetration depths [36]. Introducing the chloride diffusion coefficient in the electrolyte and effective chloride diffusion coefficient into Equation (A10) one can calculate geometry coefficients of the porous structure of a cementitious material:

(26) $$\phi /\tau^{2}=D_{\mathrm{Cl}}^{eff}/D_{\mathrm{Cl}}=6\cdot 10^{-12}\;m^{2}\;s^{-1}/2.011\cdot 10^{-9}m^{2}\;s^{-1}=2.9\cdot 10^{-3}.$$

Based on (26), the effective diffusion coefficients of sodium, potassium, and hydroxide ions were calculated. They are presented in Table 9.

### 9.2. The Influence of Different Activity Coefficient Models on Concentration Profiles and Potential Distribution

**Figure 20:**
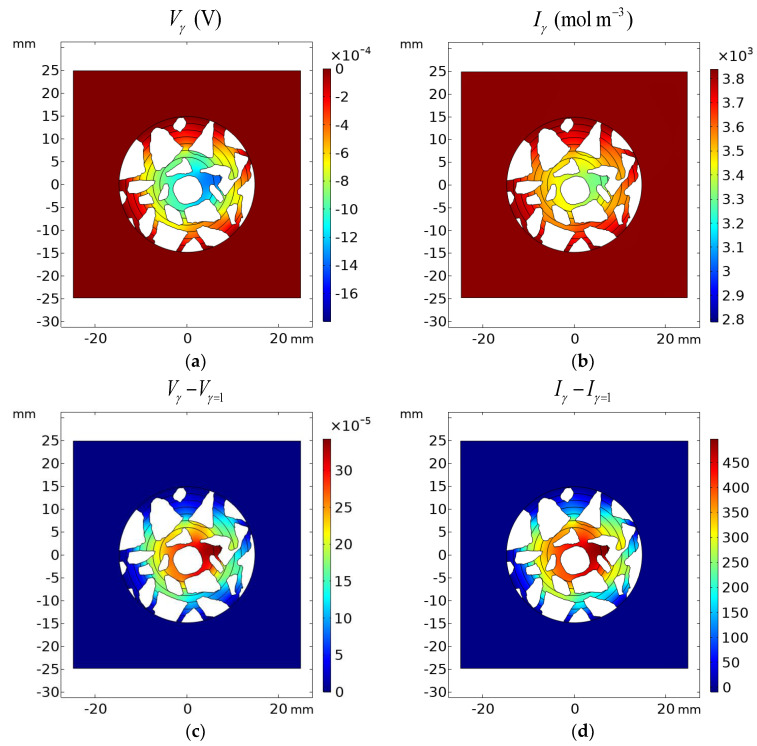
Results of calculations: (**a**) potential distribution *V_γ_* and (**b**) ionic strength *I* in the cement sample after 1200 h of immersion in 20% NaCl water solution assuming the Pitzer activity model; (**c**) deviation of the Pitzer activity model solution from the ideal solution approximation (*γ* = 1) of potential *V_γ_* − *V_γ=_*_1_ and (**d**) ionic strength *I_γ_* − *I_γ=_*_1_.

**Figure 21:**
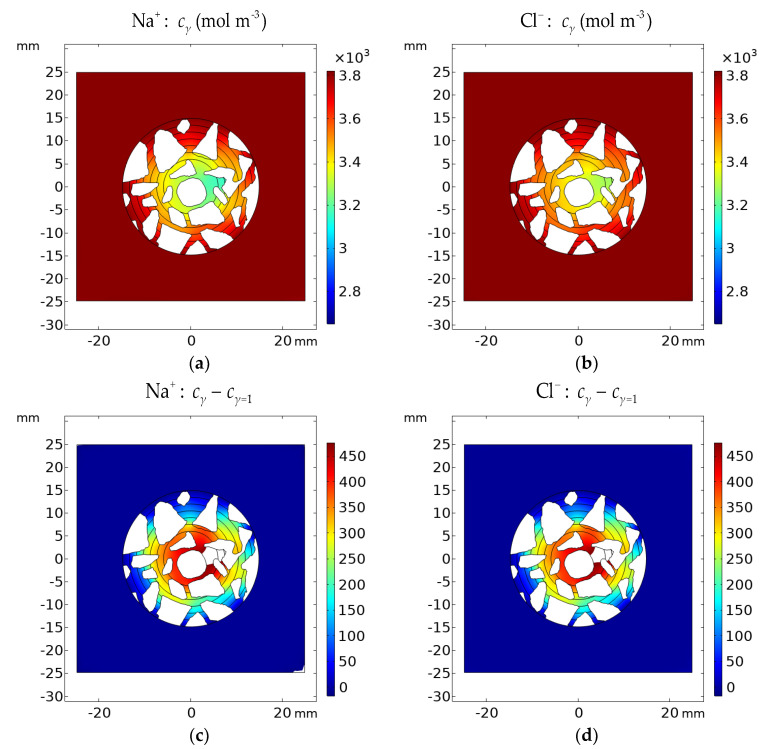
Calculated concentration distribution *c_γ_* of (**a**) Na^+^ and (**b**) Cl^−^ in the cement sample after 1200 h of immersion in 20% NaCl water solution assuming the Pitzer activity model. Deviation of the Pitzer activity model solution from the ideal solution approximation (*γ* = 1) *c_γ_* − *c_γ=_*_1_ for (**c**) Na^+^ and (**d**) Cl^−^.

**Figure 22:**
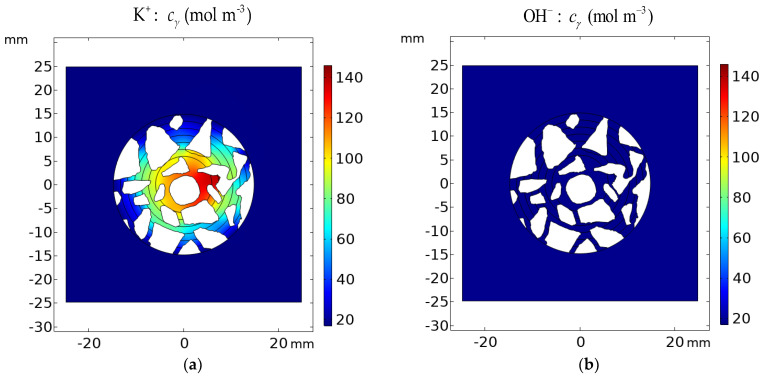

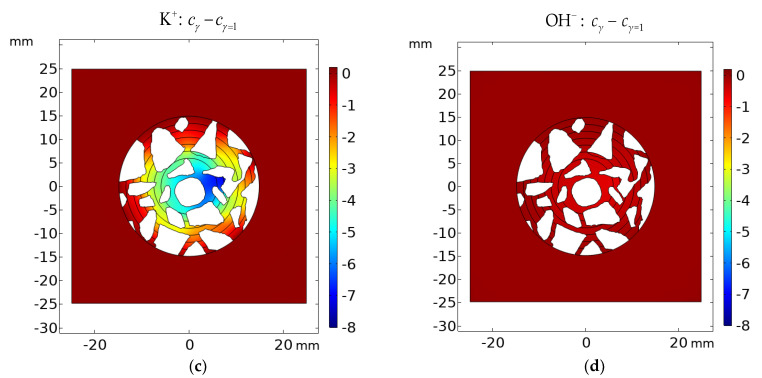
Calculated concentration distribution *c_γ_* of (**a**) K^+^ and (**b**) OH^−^ in the cement sample after 1200 h of immersion in 20% NaCl from the ideal solution approximation (*γ* = 1) *c_γ_* − *c_γ=_*_1_ for (**c**) K^+^ and (**d**) OH^−^.

Analysis of Figure 20, Figure 21 and Figure 22 shows the influence of aggregates in the concrete on the transport of ions, as well as the distribution of the potential in the concrete. One can see that the electric potential *V* and ionic strength are not uniformly distributed in the vicinity of the rod or the remaining part of the concrete sample—Figure 20. Ion concentrations are affected as well by aggregates and are not uniform in the sample—Figure 21 and Figure 22.

Figure 20, Figure 21 and Figure 22 present the difference between the Pitzer activity model and the ideal solution approximation (*γ* = 1) for potential (*V*_γ_ − *V*_γ=1_), ionic strength (*I*_γ_ − *I*_γ=1_), and concentrations $\left(c_{\gamma }-c_{\gamma =1}\right)$ of ions. These deviations for the cement sample immersed for 1200 h in 20% NaCl water solution are on the order of ~10%.

### 9.3. Fluctuations of Ion Concentration and Electrical Potential Due to the Presence of Microstructure

**Figure 23:**
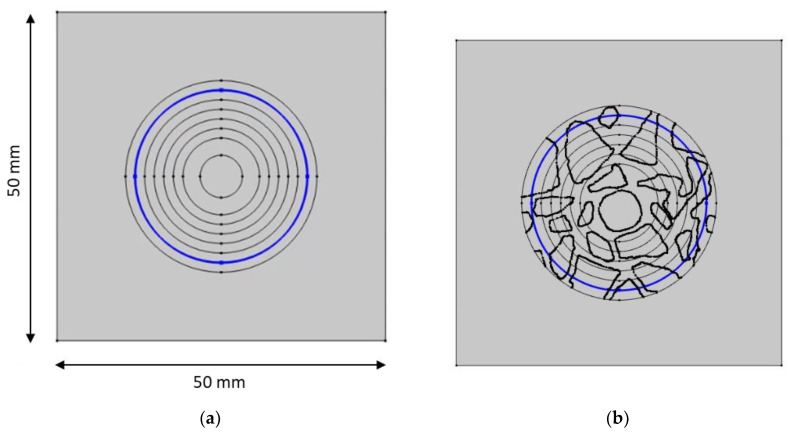

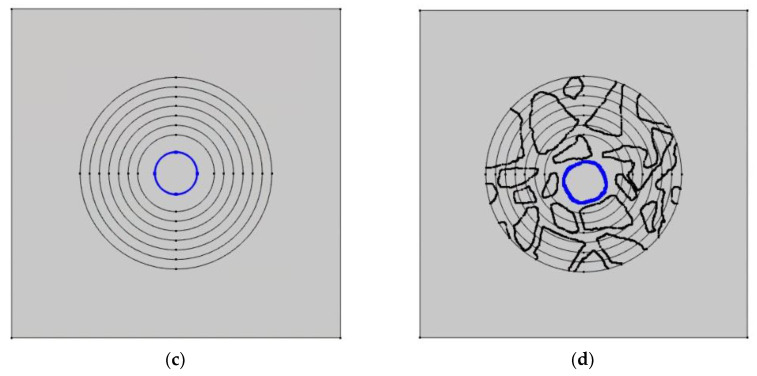
Nonhomogeneous—concrete sample—and homogeneous—mortar sample—with lines (blue) 13 mm from the center and on the surface of the rod. (**a**) mortar sample—13 mm from the center. (**b**) concrete sample—13 mm from the center. (**c**) mortar sample—the surface of the rod. (**d**) concrete sample—the surface of the rod.

To demonstrate this nonhomogeneity, the distributions of the concentration of ions, as well as the distribution of the potential and the ionic strength along a circular line at 13 mm from the sample center (Figure 23b) and on the surface of the rod (Figure 23d) in the concrete are compared with mortar samples (Figure 23a,c).

**Figure 24:**
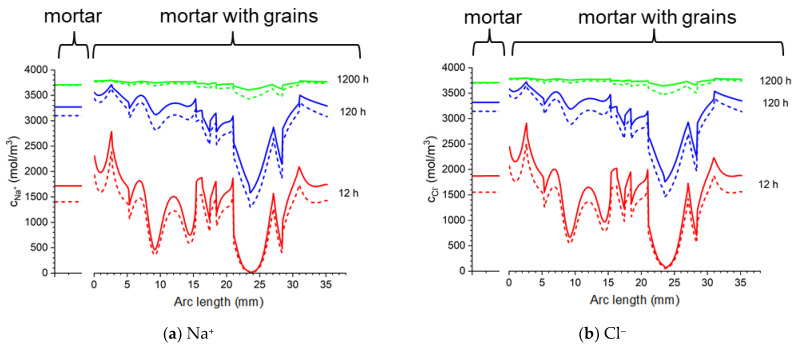

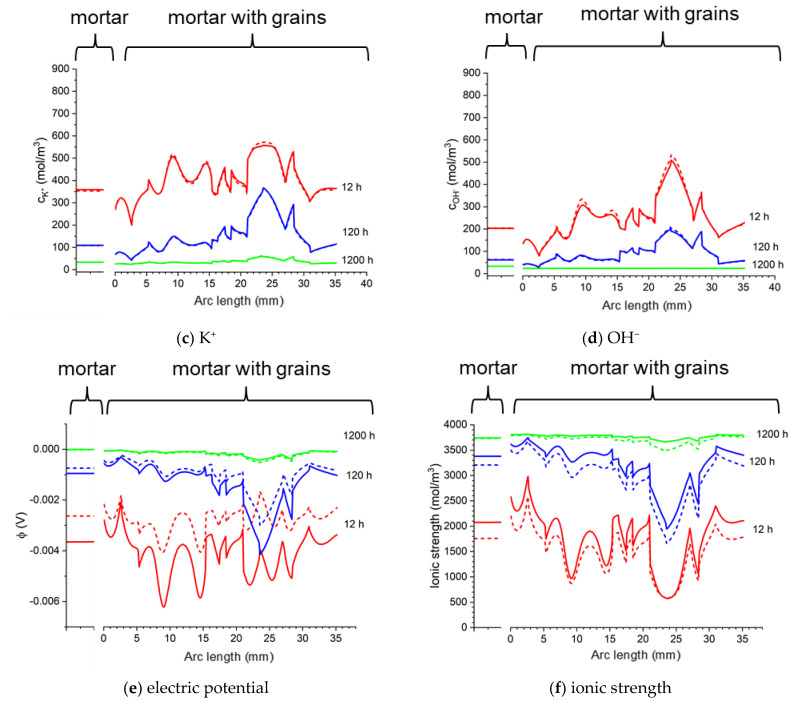
Calculated: ion concentrations: (**a**) Na^+^, (**b**) Cl^−^, (**c**) K^+^, (**d**) OH^−^, (**e**) potential (ϕ), and (**f**) ionic strength in the mortar and concrete samples at the distance 13 mm from the center of the sample in the sample in 20% NaCl water solution (along a blue line). Solid lines correspond to the solution for the Pitzer activity model and dashed lines are the solution neglecting activities (γ=1).

Figure 24 shows the concentrations of sodium, chloride, potassium, and hydroxide ions at a distance of 13 mm from the center of the concrete sample (along the blue line in Figure 23b) and at the same distance in the mortar (along the line in Figure 23a) after different times of sample immersion in 20% NaCl water solution. The solid lines show the calculations for the Pitzer model, and the dashed line is the solution for an ideal solution approximation (*γ* = 1). In the concrete sample, the ion concentrations along the line equidistant from the center of the sample differ significantly (“*mortar with aggregates*” parts in Figure 24), which is caused by the extension of the diffusion path caused by the presence of aggregate in the concrete sample. For comparison, the uniform concentrations of ions at the same distance from the center of the mortar sample are also shown (“*mortar*” parts in Figure 24).

**Figure 25:**
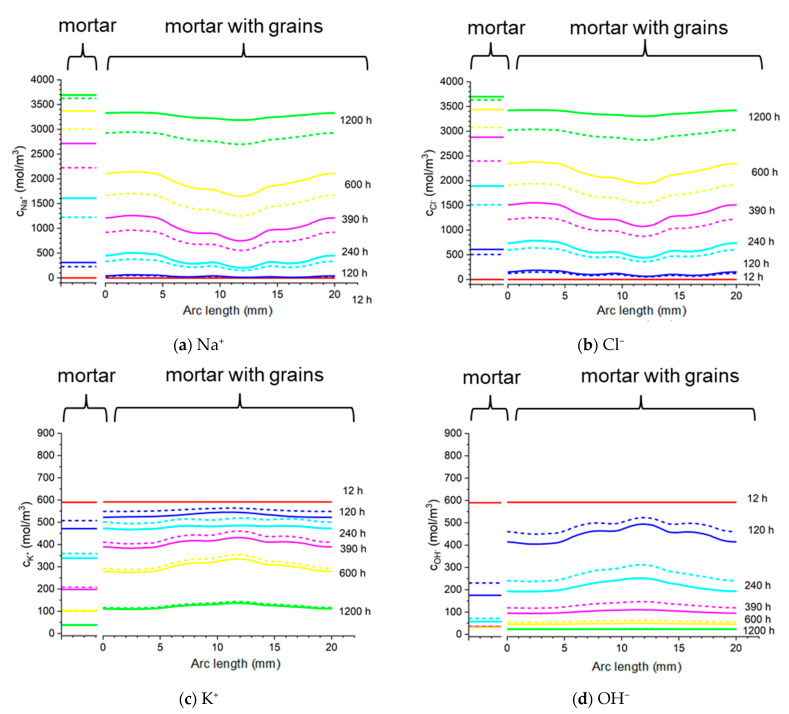

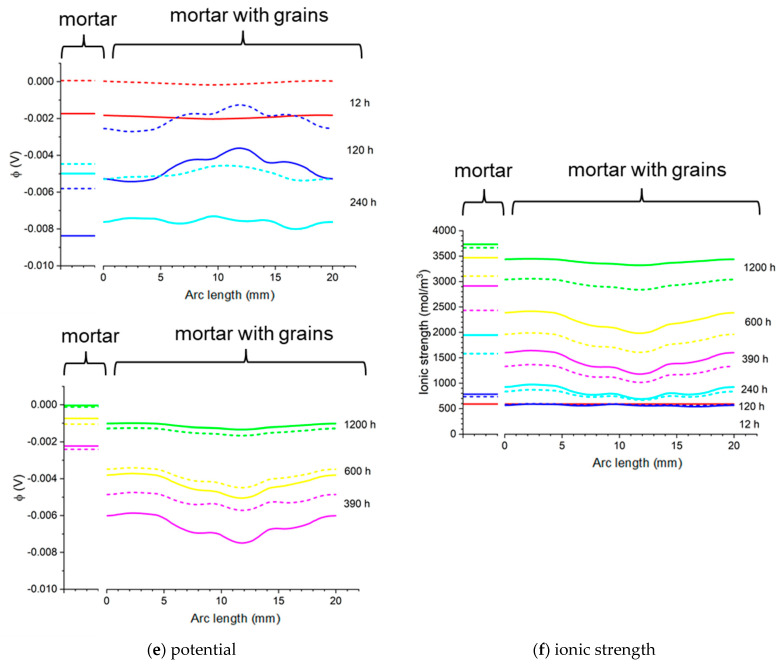
Calculated: ion concentrations: (**a**) Na^+^, (**b**) Cl^−^, (**c**) K^+^, (d) OH^−^, (**e**) potential (ϕ), and (**f**) ionic strength in the mortar and concrete samples at the surface of the rod in 20% NaCl water solution (along a blue line). Solid lines correspond to the solution for the Pitzer activity model and dashed lines are the solution neglecting activities (γ=1).

Figure 25 shows the concentrations of sodium, chloride, potassium, and hydroxide ions on the surface of the rod in the concrete sample (along the line in Figure 23d) and in the mortar (along the line in Figure 23c) after different process times of sample immersion in 20% NaCl water solution. The solid lines show the calculations for the Pitzer activity model, and the dashed line is the solution for an ideal solution approximation (*γ* =1). Ion concentrations on the surface of the rod for the mortar sample are constant (“*mortar*” parts in Figure 25), which result from the symmetry of the system and differ in value in relation to concrete samples. Inhomogeneous distribution of ion concentrations in concrete (“*mortar with aggregates*” parts in Figure 25), in particular chloride ions, when Cl^−^ threshold concentration is reached may result in a local break in the passive oxide layer and consequential development of nonuniform corrosion of the rod in concrete.

A similar trend is observed for dilute solutions and calculations for a 5% NaCl water solution are shown in Supplementary Materials S3.

## 10. Discussion

The use of the extended NPP model applied to the ionic transport in porous cementitious materials allows for the visualization of the effect of different activity models on the total flow of individual ions, their diffusion, and migration. To the best of our knowledge, for the first time emphasis has been put on the activity-induced fluxes combined with a real material’s micro-structure obtained from X-ray computed tomography (XCT).

The following assumptions and simplifications were made. The effect of double-charge ions, like Ca^2+^ or Mg^2+^, and the presence of CO_2_ were ignored. The possibility of the formation of ionic complexes was omitted. In addition, the calculations did not include the interactions of ions with the walls of the pores. Several model parameters, like the permittivity of the liquid in pores, the mobility of ions, and temperature, were assumed to be constant.

The most important aspects of the present approach are:

- a critical review of different models for the description of ion activities in concentrated multi-ion electrolytes;
- the influence of activity models (including their specific parameters) on the transport of ions and the electric potential distribution in cementitious materials, in particular, ion fluxes;
- the influence of concrete microstructure, including aggregates and porosity in 3D geometry, on the transport of ions and electric potential distribution;
- the influence of pivotal parameters of the NPP model (diffusion coefficients and dielectric permeability) on ionic transport and potential distribution in highly concentrated electrolyte solutions contained in pores.

The manuscript focuses on a quantitative description of ion transport in cementitious materials. Important differences in the ion transport rates for a mortar sample and a concrete sample (including microstructure, aggregates, and porosity) are demonstrated. The differences between ion concentration with or without activities (i.e., when the activity coefficient is equal to 1) are shown in Figure 20, Figure 21 and Figure 22. It can be observed that both the ionic strength and the potential are higher when activities are used. This is particularly pronounced close to the sample rebar. The effect of activity on the ion distribution shows that a concentration due to the ionic activity is observed for Na+ and Cl- ions. The opposite effect is noticed for the concentrations of ions initially present in the pores, i.e., K+ and OH-, although the decrease in OH- concentration is negligible. Figure 24 and Figure 25 present the influence of concrete microstructure as well as of the ionic activity on the ion concentrations at some distance from the boundary and on the surface of the reinforcement (rebar). The chloride ion concentration is not uniform, and it can vary up to 25% due to the porous nature of the sample. The solid and dashed lines in Figure 24 and Figure 25 show chloride ion concentrations with and without considering ion activities, respectively. This effect is observed for all ions at all observation times and should be taken into account when describing quantitatively ion transport, in particular the transport of chloride ions.

The quantitative description of ionic transport, including ion activities and concrete microstructure, is crucial for more accurate design and prediction of the lifetime of reinforced concrete structures. In particular, ion and oxygen concentrations, as well as the electric potential, have a substantial influence on the reinforcement corrosion reactions. The concrete’s morphology and non-homogeneous chloride ion concentration on the rod surface can lead to pitting corrosion. Also, ion activities influence the rate of chloride and other ion transport, and consequently the time of the beginning of reinforcement corrosion. As it was depicted earlier, both concrete microstructure and ion activities have a strong impact in this case. Moreover, while ion activities have rather a minor influence on ion velocity, they do influence the electric potential distribution in concrete. The same electric potential via Butler–Volmer boundary conditions has a decisive impact on the rate of corrosion reactions, and consequently on the corrosion of reinforcement.

The approach presented here allows the consideration of different porous material shapes, from flat membranes to 3D structures. The effect of the nonideality of ions is numerically shown by relating the activity defined by different models to the activity equal to the concentration of the ions. The results obtained demonstrate the quantitative link between the transport and the electrochemical potential gradient by studying the activity of ions in the porous material. The different activity models tested and evaluated demonstrate that the Pitzer model is the most reliable in the representation of mean ionic activities.

The approach outlined in the paper is still under investigation and subject to future modifications. Nevertheless, despite present idealizations, it delivers a basis capable of bringing a semblance of order and a new perspective to chemical research and applied materials chemistry.

## 11. Conclusions

The key observations from the present study can be summarized as follows:

- For the first time, different conventions and models of ion activity combined with a real material’s microstructure obtained from X-ray computed tomography were compared in their significance and influence on the ion fluxes.
- The use of the extended NPP model applied to the ionic transport driven through the activity gradient in porous cementitious materials allowed for the visualization of the effect of different activity models on the total, diffusion and migration flows of individual ions as well as ion concentrations and electric potential fields.
- The concrete microstructure strongly influences the ion concentration and the electric potential distribution with time. This effect is especially pronounced for concentrated liquid pore electrolyte solutions when ion activities are taken into account in the ion transport modeling.
- While ion activities have rather a minor influence on ion velocity, they do influence the electric potential distribution in concrete.
- The different activity models evaluated demonstrate that the Pitzer model is the most accurate in the representation of mean ionic activities.
- Despite the idealizations, the approach proposed here gives a new perspective to chemical research and applied materials chemistry. Anisotropy and pore effects in the membranes of ion-selective electrodes or diaphragms in solid fuel cells, solid contact sensors, and corrosion of reinforcing steel can be mentioned as obvious areas for the implementation of the methodology presented here.

## Figures and Tables

**Table 1:** Initial concentrations of ions in liquid in pores of cementitious material $c_{i,pore}^{0}$ (mol dm^−3^). The symbol *I* denotes molar ionic strength of the solution; $c_{2,pore}^{0}=0$ (concentration of Cl^−^ is zero).

$c_{i,pore}^{0}\;(\mathrm{mol}\;m^{-3})$	Ion 1: Na^+^	Ion 3: K^+^	Ion 4: OH^−^	*I* (mol m^−3^)
Case 1	0	590	590	590
Case 2	120	470	590	590

**Table 2:** Iinitial concentrations of ions in the external solution $c_{i,ext}^{0}$ (mol dm^−3^) for six cases studied (1, 3, 5, 10, 15, and 20% NaCl water solution). *I* denotes the molar ionic strength of the solution.

	NaCl wt %	Ion 1: Na^+^ (mol m^−3^)	Ion 2: Cl^−^ (mol m^−3^)	*I* (mol m^−3^)
Case 1	1	170	170	170
Case 2	3	510	510	510
**Case 3**	**5**	**860**	**860**	**860**
Case 4	10	1710	1710	1710
Case 5	15	2570	2570	2570
**Case 6**	**20**	**3420**	**3420**	**3420**

*Note*: due to the natural length limitations of the paper, we decided to present here Case 3 and Case 6 only. They are marked in bold. All other results can be obtained on request from the corresponding author.

**Table 3:** The usable ranges of the most popular models of activity coefficient [14], quoted after [15].

Activity Coefficient Model	Ionic Strength (mol m^−3^) Range of Application
Extended Debye–Hückel	0–100
Davies	0–600
Truesdell–Jones	0–2000
Specific Ion Interaction Theory	0–4000
Pitzer model	0–6000

**Table 4:** The parameters of the Truesdell–Jones model (see (11)) used in this work (based on [14]).

Ion *i*	$a_{i}\;(Å)\;$	$b_{i}\;({\mathrm{dm}}^{3}\;{\mathrm{mol}}^{-1})\;$
Na	4.32	0.06
Cl	3.71	0.01
K	3.71	0.01
OH (case #1)	10.65	0.21
OH (case #2)	3.5 *	0.21

* Typical value of a Debye–Hückel radius.

**Table 5:** Effective ionic radii $a_{i}$ used in Equation (12) [16].

*a_i_* (Å)				
Case	Ion 1: Na^+^	Ion 2: Cl^−^	Ion 3: K^+^	Ion 4: OH^−^
#1	3.0	3.0	3.0	3.0
#2	3.0 *	2.0 *	3.3 *	3.0 *

* Adjusted to Pitzer’s model.

**Table 6:** Mesh parameters used in calculations of ions concentration and potential distributions in the concrete sample with the corresponding time of calculations.

Mesh	No. of Elements	Avg. Element Quality	Time of Calculations
coarse	45,911	0.828	1 h 13 min
fine	71,745	0.828	1 h 47 min
finer	94,588	0.838	2 h 19 min
extra fine	126,652	0.850	3 h 33 min

**Table 7:** Calculated chloride ion concentration and electrical potential average absolute and relative errors for representative time 660 h of the process, for different meshes (see Table 6).

Mesh	Chloride Ion Concentration		Electric Potential	
	Average Absolute Error [mol m^−3^]	Average Relative Error [%]	Average Absolute Error [V]	Average Relative Error [%]
(fine)—(coarse) *	1.2	0.04	1·10^−6^	0.3
(finer)—(fine)	0.3	0.01	2·10^−7^	0.07
(extra fine)—(finer)	0.4	0.013	1.6·10^−7^	0.05

* (fine)—(coarse) should be understood as the *difference* between solutions calculated for mesh “fine” and solutions for mesh “coarse”.

**Table 8:** Parameters in Equation (23) adjusted for two-component systems [59] using the Excel solver.

Electrolyte	*a*	*b*
NaCl	0.0409	0.0008
NaOH	0.0433	0.0015
KOH	0.0479	0.0013
KCl	0.0467	0.0009

**Table 9:** Charge numbers, diffusion coefficients of ions in electrolyte $D_{i}$ and ions effective diffusion coefficients $D_{i}^{eff}$ in the liquid in pores of cement at 25 °C [36].

	Ion 1: Na^+^	Ion 2: Cl^−^	Ion 3: K^+^	Ion 4: OH^−^
*z_i_*	+1	−1	+1	−1
$D_{i}\;\;(m^{2}\;s^{-1})$	1.356·10^−9^	2.011·10^−9^	1.983·10^−9^	5.27·10^−9^
$D_{i}^{eff}\;\;(m^{2}\;s^{-1})$	3.932·10^−12^	6.0·10^−12^	5.751·10^−12^	1.528·10^−11^

**Figure A1:**
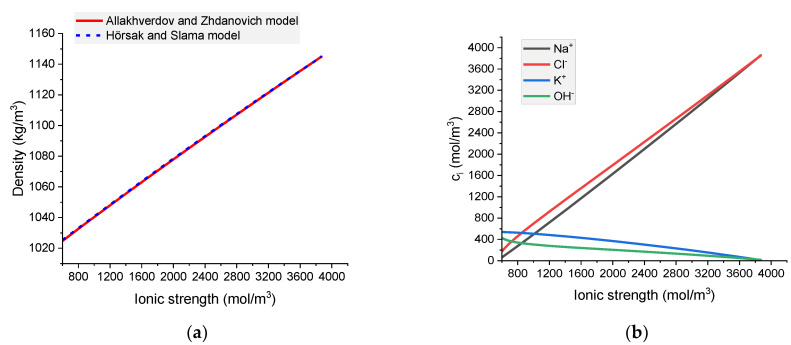
Calculated (**a**) densities of Na-K-Cl-OH water solutions using the Allakhverdov and Zhdanovich [58] and Hörsak and Slama [72] models as a function of ionic strength; and (**b**) corresponding ion concentrations as a function of ionic strength.

## Data Availability

Data is contained within the article or Supplementary Materials.

## Supplementary Materials

The following supporting information can be downloaded at: https://www.mdpi.com/article/10.3390/ma16031116/s1, Supplementary Materials S1. Contains figures showing molar individual activity coefficients of Na^+^, K^+,^ and OH^−^ ions in Na-Cl-K-OH water solutions. Figure S1. Molar individual activity coefficients in Na-Cl-K-OH water solutions as a function of ionic strength for the fixed: (a) KOH concentration 470 mol·m^−3^; and NaOH concentration 120 mol·m^−3^ and (b) KOH concentration 590 mol·m^−3^. Notations #1 and #2 correspond to different ionic radii—see Table 4 and Table 5. Supplementary Materials S2. Shows ions fluxes and their components for diluted solution (Figure S2). Figure S2. Influence of diffusion, migration, and activity terms on the total flux of Na^+^, Cl^−^, K^+,^ and OH^−^ ions in the mortar after 5 days immersion in 5 wt% NaCl water solution for Pitzer (a) and MacInnes scaling (b). Supplementary Materials S3. Compares ion concentrations and potential distribution in concrete and mortar samples for dilute solution (Figures S3–S7). Figure S3. Calculated potential distribution *V_γ_* (a) and ionic strength *I*; (b) in the cement sample after 1200 h of immersion in 20% NaCl water solution assuming the Pitzer activity model. Deviation of the Pitzer activity model solution with the ideal solution approximation (*γ* = 1) of potential *V*_γ_ − *V*_*γ*=1_ (c) and ionic strength *I_γ_* − *I*_*γ*=1_ (d). Figure S4. Calculated concentration distribution *c_γ_* of Na^+^ (a) and Cl^−^ (b) in the cement sample after 1200 h of immersion in 20% NaCl water solution assuming the Pitzer activity model. Deviation of the Pitzer activity model solution with the ideal solution approximation (*γ* =1 ) *c_γ_* − *c*_*γ*=1_ for Na^+^ (c) and Cl^−^ (d) ions. Figure S5. Calculated concentration distribution *c_γ_* of K^+^ (a) and OH^−^ (b) in the cement sample after 1200 h of immersion in 20% NaCl water solution assuming the Pitzer activity model. Deviation of the Pitzer activity model solution with the ideal solution approximation (*γ* =1 ) *c_γ_* − *c*_*γ*=1_ for K^+^ (c) and OH^−^ (d) ions. Figure S6. Calculated: ion concentrations: (a) Na^+^, (b) Cl^−^, (c) K^+^, (d) OH^−^ (e) potential (ϕ) and (f) ionic strength in the mortar and concrete samples at the distance 13 mm from the center of the sample in the sample in 5% NaCl water solution (along a blue line). Solid lines correspond to the solution for the Pitzer activity model and dashed lines are the solution neglecting activities (*γ* = 1). Figure S7. Calculated: ion concentrations: (a) Na^+^, (b) Cl^−^, (c) K^+^, (d) OH^−^, (e) potential (ϕ), and (f) ionic strength in the mortar and concrete samples at the surface of the rod in 5% NaCl water solution (along a blue line). Solid lines correspond to the solution for the Pitzer activity model and dashed lines are the solution neglecting activities (*γ* = 1).

## Appendix A

### Appendix A.1. Ionic Transport in Electrolyte Solution

A macroscopic description of the movement of ionic and non-ionic species in electrolyte solutions requires a specification of the flux $J_{i}\;(\mathrm{mol}\;\;m^{-2}\;\;s^{-1})$ for each species *i*. The expression for the flux is always to some degree an approximation of the real statistical process of mass transport in bulk. As far as electrolytes are concerned there are two basic models present in the literature to provide the flux: dilute solutions and concentrated solutions, although the latter is not fully developed. The dilute solution model is known as the Nernst–Planck equation and is based on Fick’s law of diffusion (flux is proportional to the gradient of concentration) and on the notion of ionic mobility (average constant velocity of a mobile ion under the action of the gradient of electrical potential):

(A1) $$J_{i}\;\;=\;\;-D_{i}\nabla c_{i}\;-\;\;u_{i}c_{i}\nabla \varphi ,$$

where $c_{i}$ (mol m^−3^) is the concentration, $D_{i}$ (m^2^ s^−1^) is the molecular diffusion coefficient, $u_{i}\;(m^{2}{\;s}^{-1}{\;V}^{-1})$ is the ionic mobility of species *i*, and *φ* (V) stands for the electric potential in the solution. If the relation between a diffusion coefficient and mobility (known as the Nernst–Einstein relation), $u_{i}=(z_{i}F/RT)D_{i}$ is used in (A1), then we obtain the usual form of the Nernst–Planck flux

(A2) $$J_{i}\;\;=\;\;-D_{i}\nabla c_{i}\;-\;\;D_{i}\frac{F}{RT}c_{i}\nabla \varphi ,$$

where *R* is the universal gas constant, *F* is the Faraday constant, *T* is the temperature (K), and $z_{i}$ is the charge number of species *i* ($z_{i}=0$ for neutral species).

The flux (A1) simply splits a driving force for transport into two parts: diffusion $\left(-D_{i}\nabla c_{i}\right)$ and migration $(-u_{i}c_{i}\nabla \varphi ).$ It can also be derived from a general non-equilibrium thermodynamics expression for a flux as being proportional to the gradient of electrochemical potential,

(A3) $$J_{i}=(c_{i}/RT)\nabla {\tilde{\mu }}_{i},$$

if we assume that the electrochemical potential has an idealized form

(A4) $${\tilde{\mu }}_{i}={\tilde{\mu }}_{i}^{\;0}+RT\;ln\;c_{i}+z_{i}F\varphi ,$$

where ${\tilde{\mu }}_{i}^{0}$ (J mol^−1^) is the electrochemical potential in a standard state. There are two simplifications in the formula (A4). First, using concentrations $\left(c_{i}\right)$ instead of activities $\left(a_{i}\right)$, second, an arbitrary split of ${\tilde{\mu }}_{i}$ into “chemical” and “electrical” parts. The first restriction can be overcome by applying some model of activity coefficients $\gamma_{i}$ (this approach will be adopted in the present paper). The second simplification is much harder to deal with and to the best knowledge of the authors it has not been fully resolved for multicomponent systems so far [5].

If we use the activity $a_{i}=\gamma_{i}c_{i}$ instead of $c_{i}$ in (A4), and insert ${\tilde{\mu }}_{i}$ into (A3), then we obtain

(A5) $$J_{i}\;=-D_{i}\nabla c_{i}\;-\;\;u_{i}c_{i}\nabla \varphi \;\;+\;\;D_{i}c_{i}\nabla (\ln\gamma_{i}).$$

If the expression for the flux is specified, then the mass conservation law for each species *i* can be applied to obtain partial differential equations (PDEs) for concentrations

(A6) $$\frac{\partial c_{i}}{\partial t}\;\;+\;\;\mathrm{div}\;J_{i}\;\;=\;\;R_{i},$$

where $R_{i}\;\;({\mathrm{mol}\;m}^{-3}\;\;s^{-1})$ is a reaction term which describes homogeneous reactions (in bulk) in which species *i* is consumed or produced.

In the case of charge transport in the electrolyte, we have also the electric potential *φ* as unknown, so the mass conservations and fluxes are not enough to close the system. An equation considering electrical interactions is necessary. It is usually either the electroneutrality condition or Poisson’s equation:

(A7) $$F\sum_{i}z_{i}c_{i}=0\;\;\;\;\;\mathrm{or}\;\;\;\;\;\mathrm{div}\left(-\varepsilon_{r}\varepsilon_{0}\nabla \varphi \right)=F\sum_{i}z_{i}c_{i},$$

where $\varepsilon_{0},\;\;\varepsilon_{r}$ are the vacuum permittivity and relative permittivity, respectively. The sum is over all charged species. In the present paper, we will use the Poisson equation for computing the electric potential.

### Appendix A.2. Ionic Transport in Porous Materials

A commonly used approach is to treat a porous material as a continuum, without explicitly separating it into the pore space and the solid skeleton. Thus, macroscopic concentration, flux, and electric potential are defined at every point x in space occupied by the material. However, these quantities are defined as the averages over a representative elementary volume. For example, the macroscopic concentration $c_{i}(x,t)$ is the microscopic concentration $c_{i}^{mic}(\xi ,t)$ over such volume. The same applies to the macroscopic φ  (x,t) electric potential:

(A8) $$\begin{array}{l} c(x,t)=\frac{1}{\left|P_{V_{x}}\right|}\int_{P_{V_{x}}}c_{i}^{mic}(\xi ,t)d^{3}\xi ,\;\\ \varphi (x,t)=\frac{1}{\left|P_{V_{x}}\right|}\int_{P_{V_{x}}}\varphi_{i}^{mic}(\xi ,t)d^{3}\xi , \end{array}$$

where the domain (usually a ball) $V_{x}$ is centered at a point x and $P_{V_{x}}$ is the pore part of $V_{x}.$ By the homogenization method it can be shown that under some assumptions the mass balance equation with the Nernst–Planck flux and no reactions takes the form [33]

(A9) $$\phi \frac{\partial c_{i}}{\partial t}\;\;=\;\;\mathrm{div}\;D_{i}^{eff}\left(\nabla c_{i}+\frac{z_{i}F}{RT}c_{i}\nabla \varphi \right),$$

where $\phi =\;\;|P_{V}|/|V|$ is the porosity and $D_{i}^{eff}$ is the effective diffusion tensor given by some integral formulae and solution to the Laplace equation with special boundary conditions. At present we cannot apply the exact transport equation such as (A9) because it is not feasible to solve for an effective diffusion matrix $D_{i}^{eff}$ for real pore systems. In practice, we approximate $D_{i}^{eff}=D_{i}^{eff}I$ where **I** is the identity matrix and $D_{i}^{eff}$ is the effective diffusion coefficient

(A10) $$D_{i}^{eff}=\phi D_{i}/\tau^{2}.$$

Equation (A10) is a modified molecular diffusion coefficient by two empirical parameters that characterize a porous medium: the already mentioned porosity (ϕ) and tortuosity (τ). The first parameter takes into account the reduction of available space for species movement. The second is related to the fact that in the network of pores, the available paths for mobile species are usually much longer than the geometrically shortest distance between any to locations. In other words, it is a measure of the elongation of the transport path (due to the porous structure) with respect to a straight line. Thus, the tortuosity is the ratio of average pore length $l_{p}$ to the length of the porous medium l along the major diffusion axis, $\tau =(l_{p}/l).$ Since $l_{p}>l$ then *τ* > 1. (Some authors use the definition $\tau ={\left(l_{p}/l\right)}^{2};$ then Equation (A10) reads as $D_{i}^{eff}=\phi D_{i}/\tau ).$

## Appendix B

### X-ray Computed Tomography (XCT) Measurements

The XCT measurements were performed using a Nanotom 180S (GE Sensing & Inspection Technologies GmbH, Wunstorf, Germany). The machine is equipped with a nanofocus X-ray tube with a maximum voltage of 180 kV. The tomograms were registered on a Hamamatsu 2300 × 2300-pixel detector. During the measurements, a tungsten target was used. The polychromatic beam was filtered using a 0.5 mm copper filter. The working parameters of the X-ray tube were I = 250 µA and V = 70 kV. A total of 1600 projections were taken with four integrations for each exposition. The total time of measurement was around 120 min. The reconstructions of the measured objects were done with the aid of the proprietary GE software, datosX ver. 2.1.0, using the Feldkamp algorithm for cone beam X-ray CT [60]. The final resolution of the reconstructed object was 16 µm. The post-reconstruction data treatment was performed using VGStudio Max 2.1 software (Volume Graphic GmbH, Heidelberg, Germany) [61].

## Appendix C

### Appendix C.1. The Theory of Debye–Hückel and Its Modifications

The ground-breaking ion-cloud theory of ion–ion interactions by Debye–Hückel was developed in 1923 [62] for very dilute aqueous solutions of strong electrolytes. An abbreviated derivation can be found in [7]. The resulting Debye–Hückel limiting law (DHLL) has the form:

(A11) $$\begin{array}{l} \log\;\;\gamma_{i}\;\;\;=\;\;-Az_{i}^{2}\sqrt{I}\;\mathrm{and}\;\log\;\;\gamma_{\pm }\;\;\;=\;\;-Az_{+}z_{-}\sqrt{I},\\ A=\frac{\sqrt{2}e_{0}F^{2}}{\ln(10)8\pi {\left(\varepsilon \varepsilon_{0}RT\right)}^{3/2}},\;I=\frac{1}{2}\sum_{i}z_{i}^{2}c_{i}. \end{array}$$

For aqueous solutions at 25 °C, the Debye–Hückel constant *A*= 0.512 mol^−1/2^ dm^1/2^, the ionic strength, *I,* is in molarity units, as well as the ionic concentrations, $c_{i}.$ Pobelov [7] states that “*the only well-established, universally agreed-upon tool providing us information about the thermodynamic properties of individual ions in solution is the Debye–Hückel limiting law*”.

Equation (A11) contains parameters defining the temperature and the average properties of the solvent (*ε*, *I*), and the only specific characteristic of an ion is its charge. Thus, it predicts that in a 1:1 electrolyte, the value of an activity coefficient depends only on the ionic strength of the solution and that the SIACs for the cation and anion are equal. At 25 °C, DHLL is valid for dilute aqueous solutions of strong electrolytes up to the ionic strength of 0.001 M. Claims of its validity up to 0.01 M, should be treated with caution [7].

Through the years, many modifications of the DHLL were developed with the purpose to extend its range of validity. The first modification relaxes the assumption of point-like ions and considers them as having a finite diameter. Then, the same line of derivation as in the original DHLL gives:

(A12) $$\log\;\;\gamma_{\pm }\;\;=\;\;-\frac{A|z_{+}z_{-}|\sqrt{I}}{1+Ba\sqrt{I}},\;\;\;\;\;B\;\;=\;\;\sqrt{\frac{2F^{2}}{\varepsilon \varepsilon_{0}RT}}.$$

For water at 25 °C, B = 3.289 mol^−1/2^·dm^1/2^·nm^−1^ [7] and *a* is an adjustable parameter in the range of 0.3–0.5 nm. This equation can produce a good fit of experimental data up to 0.1 M. It is often referred to as the extended Debye–Hückel equation.

The Bates–Guggenheim [63,64] equation, which is the corner stone of the IUPAC definition of pH, assumes a = 0.456 nm to give the form:

(A13) $$\log\;\;\gamma_{i}\;\;=-\frac{Az_{i}^{2}\sqrt{I}}{1+1.5\sqrt{I}}.$$

Examination of experimental values of MIACs shows that generally, they decrease to minimal values at lower ionic strengths, and then increase at higher ionic strengths. However, the form of Equation (A12) is such that it predicts $\gamma_{\pm }$ values that decline continuously with ionic strength. To extend the applicability of Equation (A13) to higher ionic strength, a linear term was added [65]:

(A14) $$\log\;\;\gamma_{\pm }\;=-\frac{A\left|z_{+}z_{-}\right|\sqrt{I}}{1+Ba\sqrt{I}}\;\;+\;\;C\cdot I.$$

This equation is known as Truesdell–Jones equation; *a* and *C* parameters are determined from experimental data. A good fit to experimental data is observed up to 2 M [14].

A much simpler version of this equation, characterized by a lack of free parameters and its mathematical simplicity, is known as the Davies equation [66].

For practical applications, an excellent review paper [67] recommends two basic approaches, which are endorsed by the OECD Nuclear Energy Agency (NEA) [68].

### Appendix C.2. The Specific Ion Interaction Theory (SIT)

SIT, also known as the Brønsted–Guggenheim–Scatchard model, was built by modifying the equation of the Debye–Hückel limiting law (DHLL) and by adding additional terms to the Debye–Hückel expression:

(A15) $$\ln\;\gamma_{i}=-\frac{z_{i}^{2}A\sqrt{I_{m}}}{1+1.5\sqrt{I_{m}}}\;\;+\;\;\sum_{k}\varepsilon (i,k)\;m_{k}.$$

Conventionally, *I* is given in units of molality, $I_{m}=\frac{1}{2}\sum_{i}z_{i}^{2}m_{i}.$ The value of *A* and the parameter 1.5 depends on the units of *I*, as the left-hand side of Equation (A15) is dimensionless. They also depend on temperature and the type of solvent used. The first term of this equation represents one of the modifications of the Debye–Hückel expression approximating the long-range interactions in a very diluted strong electrolyte solution. The second accounts for the middle and short-range effects occurring at higher concentrations.

### Appendix C.3. Pitzer’s Equations

These are a virial expansion of the Gibbs free energy [69] and they consider the interactions between the ions and the solvent, as well as between the ions themselves. Therefore, they are particularly useful in calculating activity coefficients in electrolyte mixtures.

The main difference between the SIT theory and the Pitzer equations lies in the fact that SIT equations do not consider the interactions between ions in the multicomponent systems, while the structure of the Pitzer equations allows the incorporation of the interactions parameters of the second and third order. The real advantage of the Pitzer model shows in calculations for the highly concentrated mixtures of electrolytes. In the range of calculations where parameters are available, they give excellent, reliable results. The U.S. National Institute of Standards and Technology (NIST) used them for the description of the thermodynamic properties of NaCl(*aq*) [70] and KCl(*aq*) [71].

For a more detailed comparison of the strengths and shortcomings of both approaches, interested readers should consult the NEA recommendations [68] and the review by May and Rowland [67].

## Appendix D

### Pitzer Equations for a Quaternary Na-Cl-K-OH System

For the system NaCl-KOH in water the Pitzer Equations (14) and (15) take the form:

$$X={\mathrm{Cl}}^{-},\;\;c\in \{{\mathrm{Na}}^{+},\;\;\;K^{+}\},\;\;a\in \{{\mathrm{Cl}}^{-},\;\;{\mathrm{OH}}^{-}\}:$$

(A16) $$\begin{array}{l} \ln\gamma_{Cl}=z_{Cl}^{2}F+m_{Na}(2B_{Na}{}_{Cl}+ZC_{NaCl})+\;m_{K}(2B_{KCl}+ZC_{KCl})+\\ m_{Cl}(2\Phi_{ClCl}+m_{Na}\psi_{NaClCl}+m_{K}\psi_{KClCl})+\;m_{OH}(2\Phi_{ClOH}+m_{Na}\psi_{NaClOH}+m_{K}\psi_{KClOH})+\\ m_{Na}m_{K}\psi_{NaKCl}+\;\;|z_{Cl}|(m_{Na}m_{Cl}C_{NaCl}+m_{Na}m_{OH}C_{NaOH}+m_{K}m_{Cl}C_{KCl}+m_{K}m_{OH}C_{KOH}) \end{array}$$

$$X={\mathrm{OH}}^{-},\;\;c\in \{{\mathrm{Na}}^{+},\;\;\;K^{+}\},\;\;a\in \{{\mathrm{Cl}}^{-},\;\;{\mathrm{OH}}^{-}\}:$$

(A17) $$\begin{array}{l} \ln\gamma_{OH}=z_{OH}^{2}F+m_{Na}(2B_{Na}{}_{OH}+ZC_{NaOH})+\;m_{K}(2B_{KOH}+ZC_{KOH})+\\ m_{OH}(2\Phi_{OHOH}+m_{Na}\psi_{NaOHOH}+m_{K}\psi_{KOHOH})+\;m_{Cl}(2\Phi_{OHCl}+m_{Na}\psi_{NaOHCl}+m_{K}\psi_{KOHCl})+\\ m_{Na}m_{K}\psi_{NaKOH}+\;|z_{OH}|(m_{Na}m_{OH}C_{NaOH}+m_{Na}m_{Cl}C_{NaCl}+m_{K}m_{OH}C_{KOH}+m_{K}m_{Cl}C_{KCl}) \end{array}$$

$$M={\mathrm{Na}}^{+},\;\;c\in \{{\mathrm{Na}}^{+},\;\;\;K^{+}\},\;\;a\in \{{\mathrm{Cl}}^{-},\;\;{\mathrm{OH}}^{-}\}:$$

(A18) $$\begin{array}{l} \ln\gamma_{Na}=z_{Na}^{2}F+m_{Cl}(2B_{Na}{}_{Cl}+ZC_{NaCl})+\;m_{OH}(2B_{NaOH}+ZC_{NaOH})+\\ m_{Na}(2\Phi_{NaNa}+m_{Cl}\psi_{NaNaCl}+m_{OH}\psi_{NaNaOH})+\;m_{K}(2\Phi_{NaK}+m_{Cl}\psi_{NaKCl}+m_{OH}\psi_{NaKOH})+\\ m_{Cl}m_{OH}\psi_{NaClOH}+\;|z_{Na}|(m_{Na}m_{Cl}C_{NaCl}+m_{Na}m_{OH}C_{NaOH}+m_{K}m_{Cl}C_{KCl}+m_{K}m_{OH}C_{KOH}) \end{array}$$

$$M=K^{+},\;\;c\in \{{\mathrm{Na}}^{+},\;\;\;K^{+}\},\;\;a\in \{{\mathrm{Cl}}^{-},\;\;{\mathrm{OH}}^{-}\}:$$

(A19) $$\begin{array}{l} \ln\gamma_{K}=z_{K}^{2}F+m_{Cl}(2B_{K}{}_{Cl}+ZC_{KCl})+\;m_{OH}(2B_{KOH}+ZC_{KOH})+\\ m_{K}(2\Phi_{KK}+m_{Cl}\psi_{KKCl}+m_{OH}\psi_{KKOH})+\;m_{Na}(2\Phi_{KNa}+m_{Cl}\psi_{KNaCl}+m_{OH}\psi_{KNaOH})+\\ m_{Cl}m_{OH}\psi_{KClOH}+\;|z_{K}|(m_{K}m_{Cl}C_{KCl}+m_{K}m_{OH}C_{KOH}+m_{Na}m_{Cl}C_{NaCl}+m_{Na}m_{OH}C_{NaOH}) \end{array}$$

(A20) $$\begin{array}{l} F=-A^{\phi }\left[\frac{\sqrt{I}}{1+b\sqrt{I}}+\frac{2}{b}\ln(1+b\sqrt{I})\right]+m_{Na}m_{Cl}{B^{\prime }}_{NaCl}+m_{Na}m_{OH}{B^{\prime }}_{NaOH}+\\ m_{K}m_{Cl}{B^{\prime }}_{KCl}+m_{K}m_{OH}{B^{\prime }}_{KOH}+m_{Na}m_{K}{\Phi^{\prime }}_{NaK}+m_{Cl}m_{OH}{\Phi^{\prime }}_{ClOH} \end{array}$$

where:

(A21) $$\begin{array}{l} B_{NaCl}=\beta_{NaCl}^{\left(0\right)}+\beta_{NaCl}^{\left(1\right)}\;g(\alpha_{NaCl}\sqrt{I}),\;\;B_{KCl}=\beta_{KCl}^{\left(0\right)}+\beta_{KCl}^{\left(1\right)}\;g(\alpha_{KCl}\sqrt{I}),\\ B_{NaOH}=\beta_{NaOH}^{\left(0\right)}+\beta_{NaOH}^{\left(1\right)}\;g(\alpha_{NaOH}\sqrt{I}),\;\;\;B_{KOH}=\beta_{KOH}^{\left(0\right)}+\beta_{KOH}^{\left(1\right)}g(\alpha_{KOH}\sqrt{I}),\\ g(s)=2(1-(1+s)e^{-s})/s^{2},\\ g^{\prime }(s)=-2\left(1-(1+s+\frac{1}{2}s^{2})e^{-s}\right)/s^{2},\\ \mathrm{where}\;s=\alpha_{MX}\sqrt{I} \end{array}$$

When either cation M or anion X is univalent, $\alpha_{MX}=2.0$.

The coefficients $\beta_{MX}^{0},\;\beta_{MX}^{1}$ and $C_{MX}^{\phi }$ are taken from [69] (Table 2, p.100)

(A22) $$\begin{array}{l} C_{NaCl}=\frac{c_{NaCl}^{\phi }}{2\sqrt{\left|z_{Na}z_{Cl}\right|}}=\frac{0.00127}{2\sqrt{\left|1\cdot (-1)\right|}}=0.000635,\;\\ C_{KOH}=\frac{c_{KOH}^{\phi }}{2\sqrt{\left|z_{K}z_{OH}\right|}}=\frac{0.0041}{2\sqrt{\left|1\cdot (-1)\right|}}=0.00205,\\ C_{KCl}=\frac{c_{KCl}^{\phi }}{2\sqrt{\left|z_{K}z_{Cl}\right|}}=\frac{-0.00084}{2\sqrt{\left|1\cdot (-1)\right|}}=-0.00042,\;\\ C_{NaOH}=\frac{c_{NaOH}^{\phi }}{2\sqrt{\left|z_{Na}z_{OH}\right|}}=\frac{0.0044}{2\sqrt{\left|1\cdot (-1)\right|}}=0.0022, \end{array}$$

(A23) $$\psi_{NaClCl}=\psi_{KClCl}=\psi_{NaOHOH}=\psi_{KOHOH}=\;\psi_{NaNaCl}=\psi_{NaNaOH}=\psi_{KKCl}=\psi_{KKOH}=0$$

(A24) $$\psi_{NaClOH}=\psi_{NaOHCl},\;\;\psi_{KClOH}=\psi_{KOHCl},\;\;\psi_{NaKCl}=\psi_{KNaCl},\;\;\psi_{NaKOH}=\psi_{KNaOH},$$

(A25) ΦClCl=ΦOHOH=ΦNaNa=ΦKK=0,ΦClOH=ΦOHCl=θClOH+θEClOH(I),ΦNaK=ΦKNa=θNaK+θENaK(I),

(A26) B′NaCl=βNaCl(1)g′(2I)/I,   B′KOH=βKOH(1)g′(2I)/I,B′NaOH=βNaOH(1)g′(2I)/I,   B′KCl=βKCl(1)g′(2I)/I,Φ′ClOH=θ′EClOH(I),   Φ′NaK=θ′EClOH(I),

The values of parameters used in the Pitzer model are taken from [69] and are presented in Table A1, Table A2 and Table A3.

To calculate θEij, which appears in (A25) and (A26), we must resort to details of the Pitzer model. It turns out that

(A27) θEij=zizj4IJ(xij)−12J(xii)−12J(xjj),θ′Eij=−θEijI+zizj8I2xijJ′(xij)−12xiiJ′(xii)−12xjjJ′(xjj),

with $x_{ij}=6z_{i}z_{j}A_{\phi }\sqrt{I}$ and *J*(*x*) some universal function:

$J(x)=\frac{1}{x}\int_{0}^{\infty }\left(1-(x/y)e^{-y}+(x^{2}/2y^{2})e^{-2y}-e^{-(x/y)e^{-y}}\right)y^{2}dy$.

This function can be evaluated numerically and tabulated, but for the special case of ions of the same charges (e.g., Na^+^, K^+^) we see from (A27) that θEij=0,  θ′Eij=0. It means that in (A25), $\Phi_{ClOH}=\Phi_{OHCl}=const$ and in (A26), ${\Phi^{\prime }}_{ClOH}={\Phi^{\prime }}_{NaK}=0.$

**Table A1:** Pitzer model parameters for Equations (15) and (22) at 25 °C.

Parameter		
$A^{\phi }$	*b*	*α*
0.3915	1.2	2

**Table A2:** Pitzer model binary parameters for Equations (23) and (24). [69], Table 2, p.100.

Index, *x*	Parameter		
	$\beta_{x}^{\left(0\right)}$	$\beta_{x}^{\left(1\right)}$	$C_{x}^{\phi }$
NaCl	0.0765	0.2664	0.00127
KCl	0.04835	0.2122	−0.00084
NaOH	0.0864	0.253	0.0044
KOH	0.1298	0.320	0.0041

**Table A3:** Pitzer model third-order parameters for Equations (26), (A1), and (A2). [69], Table 18, p.118.

Index *x*	Parameter	Index *x*	Parameter		
	$\psi_{x}$		$\theta_{x}$	θ ExI	θ′ExI
NaClOH	−0.006	ClOH	0	−0.050	0
KClOH	−0.006				
NaKCl	−0.0018	NaK	0	−0.012	0
NaKOH	−0.0018				

## Appendix E

### Molal and Molar Activity Coefficients

The molal activity coefficient, $\gamma_{i},$ is defined as:

(A28) $$a_{i}(m)=m_{i}/m_{0}\gamma_{i},\;\left[m_{i}\right]=m_{i}\left[\frac{\mathrm{mol}}{{\mathrm{kg}}_{solvent}}\right],\;\left[m_{0}\right]=1\left[\frac{\mathrm{mol}}{{\mathrm{kg}}_{solvent}}\right]$$

where *a_i_*(*m*), *m_i_* are the molal activity and molality of *i*-th component, respectively; and *m*_0_ is 1 unit molality.

Analogically, the molar activity coefficient, $y_{i},$ is defined as:

(A29) $$a_{i}(c)=c_{i}/c_{0}y_{i},\;\left[c_{i}\right]=c_{i}\left[\frac{\mathrm{mol}}{m^{3}}\right],\;\left[c_{0}\right]=1\left[\frac{\mathrm{mol}}{m^{3}}\right]$$

where $a_{i}(c),\;\;c_{i}$ are the molar activity and molarity of *i*-th component, respectively; and *c*_0_ is 1 unit molarity.

Because values of molal and molar activities are equal [9]

(A30) $$a_{i}(m)=a_{i}(c)$$

consequently, the values of the activity coefficients are different and depend on the assumed unit of concentration: molality or molarity.

Thus, the molal activity $\gamma_{i}$ is related to the molar activity, *y*_i_ by the relation:

(A31) $$y_{i}=\frac{m_{i}}{c_{i}}\frac{c_{0}}{m_{0}}\gamma_{i}=\frac{m_{i}}{c_{i}}\frac{1}{d_{0}}\gamma_{i}$$

where *d*_0_ is the density of the solvent.

## Appendix F

### Hörsak and Slama Density Model for Multicomponent Solutions

The model proposed by Hörsak and Slama has been extended to multicomponent solutions [72]

(A32) $$\rho (x_{1},\ldots ,x_{N_{ION}})=\frac{\sum_{i=1}^{N_{ION}}x_{i}M_{i}+x_{w}M_{w}}{\sum_{i=1}^{N_{ION}}\nu_{i}^{o}\cdot x_{i}+\nu_{w}^{o}\cdot x_{w}+x_{w}\sum_{i=1}^{N_{ION}}\alpha_{i}\cdot x_{i}}$$

$n_{w}=1000\;g/M_{w}=55.51\;\;\mathrm{mol}$ (number of water moles per liter), *n_i_*—molality of *i*-th ion in solution, the total number of moles $n=n_{w}+\sum_{i=1}^{N_{ION}}n_{i},$ $x_{i}=n_{i}/n$—the molal fraction of *i*-th ion, $x_{w}=1-\sum_{i=1}^{N_{ION}}x_{i}$, $M_{i}$—molecular weight of ions, $M_{w}$—molecular weight of water, $N_{ION}$—the number of moles in solution with (*i*) representing each ion, with (*w*) as the solvent, water; $\nu_{w}^{o}$—molar volume of water.

The equation can be applied to aqueous systems containing *n* ions in the solutions, requiring the values for $N_{ION}$ parameters of $\nu_{i}^{o}$ and $N_{ION}$ parameters for α*_i_* for the prediction, which can be obtained beginning with the information on the densities of binary aqueous systems, that is, two ions in solution ($N_{ION}=2$).

For *m*_NaCl_ molal NaCl and *m*_KOH_ molal KOH aqueous solution, we have:

(A33) $$\begin{array}{l} n_{Na}=m_{NaCl},\;\;\;n_{Cl}=m_{NaCl},\;\;\;n_{K}=m_{KOH},\;\;\;n_{OH}=m_{KOH},\\ n=n_{Na}+n_{Cl}+n_{K}+n_{OH}+n_{w}=2m_{NaCl}+2m_{KOH}+n_{w},\\ x_{Na}=x_{Cl}=\frac{m_{NaCl}}{n},\;\;x_{K}=x_{OH}=\frac{m_{KOH}}{n},\;\;x_{w}=1-x_{Na}-x_{Cl}-x_{K}-x_{OH}. \end{array}$$

And the density of the solution equals:

(A34) $$\begin{array}{l} \rho (x_{Na},x_{Cl},x_{K},x_{OH})=\\ =\frac{(x_{Na}M_{Na}+x_{Cl}M_{Cl}+x_{K}M_{K}+x_{OH}M_{OH})+x_{w}M_{w}}{\left(x_{Na}\nu_{Na}^{o}+x_{Cl}\nu_{Cl}^{o}+x_{K}\nu_{K}^{o}+x_{OH}\nu_{OH}^{o})+\nu_{w}^{o}\cdot x_{w}+x_{w}\cdot (x_{Na}\alpha_{Na}+x_{Cl}\alpha_{Cl}+x_{K}\alpha_{K}+x_{OH}\alpha_{OH}\right)}. \end{array}$$

**Table A4:** Parameters for calculation of ionic densities at 298.15 K [72].

Ion	$\nu_{i}^{o}({\mathrm{cm}}^{3}\;{\mathrm{mol}}^{-1})$	$\alpha_{i}({\mathrm{cm}}^{3}\;{\mathrm{mol}}^{-1})$
Na^+^	14.6883	−5.9081
Cl^−^	23.5130	−14.6221
K^+^	27.0279	−8.0763
OH^−^	17.2940	−29.3781

In Figure A1 comparison of densities calculated based on Allakhverdov and Zhdanovich [58]; and Hörsak and Slama [72] models for various ionic strengths and the solution compositions are presented. Both models give very similar results in a wide range of concentrations (ionic strength).
