# Supplementary material for: The Influence of Chemical Activity Models on the Description of Ion Transport through Micro-Structured Cementitious Materials
Source: Materials (Basel). 2023 Jan 28;16(3):1116. doi: 10.3390/ma16031116 (PMC9920105; doi:10.3390/ma16031116)
Supplement: Supplementary file 1 [file materials-16-01116-s001.zip › materials-2145291-SI.pdf]

The Influence of Chemical Activity Models on the Description of Ion Transport through Micro-Structured Cementitious Materials

Krzysztof Szyszkiewicz-Warzecha [1], Grażyna Wilczek-Vera [2], Andrzej Lewenstam [1], Anna Górska [1], Jacek Tarasiuk [1] and Robert Filipek [1,*]

[1] AGH University of Science and Technology, Faculty of Materials Science and Ceramics, Al. Mickiewicza 30, 30-059 Kraków, Poland; szyskin@agh.edu.pl (K.S.-W.); alewenst@agh.edu.pl (A.L.); anna.chyba@student.agh.edu.pl (A.G.); tarasiuk@agh.edu.pl (J.T.)

[2] McGill University, Departament of Chemistry, 845 Sherbrooke Street West Montreal, Quebec H3A 0G4, Canada; grazyna.wilczek@mcgill.ca

* Correspondence: rof@agh.edu.pl

Supplementary Materials

Supplementary Material 1

Molar individual activity coefficients of Na^+, K^+ and OH^- ions in Na-Cl-K-OH water solutions.

Na-Cl-K-OH water solutions with the fixed 120 mol·m^{-3} NaOH and 470 mol·m^{-3} KOH concentrations (a)	Na-Cl-K-OH water solutions with the fixed 590 mol·m^{-3} KOH concentration b)

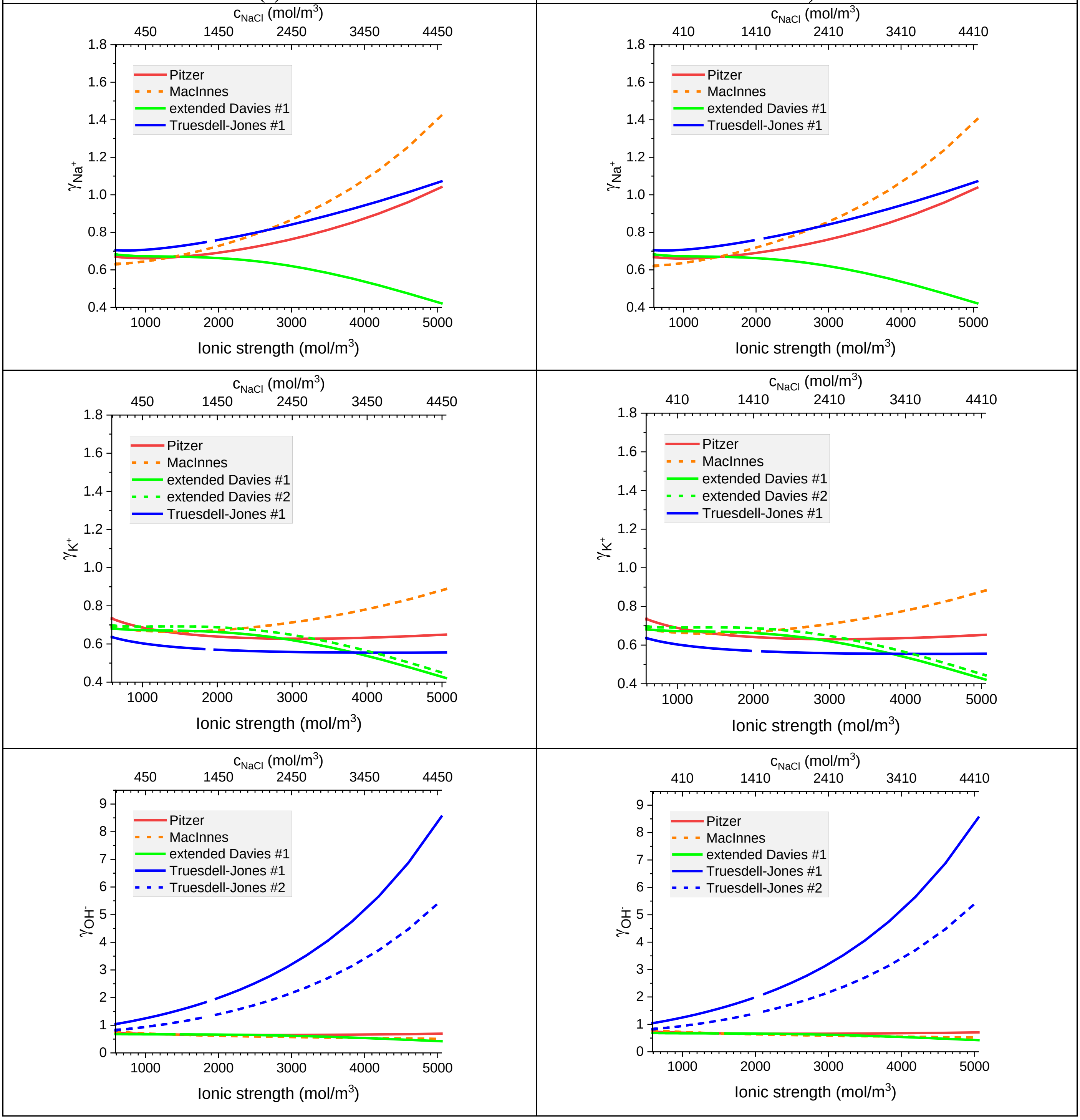

Figure S1. Molar individual activity coefficients in Na-Cl-K-OH water solutions as a function of ionic strength for the fixed: a) KOH concentration 470 mol·m^{-3}; and NaOH concentration120 mol·m^{-3}and b) KOH concentration 590 mol·m^{-3}. Notations #1 and #2 correspond to different ions radiuses – see Table 4 and Table 5.

Supplementary Material 2

Comparison of ions fluxes and their components for diluted solution.

Pitzer model<br>a)	MacInnes scaling<br>b)
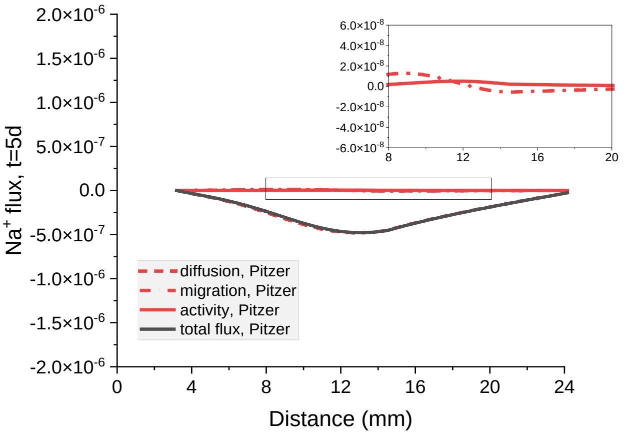	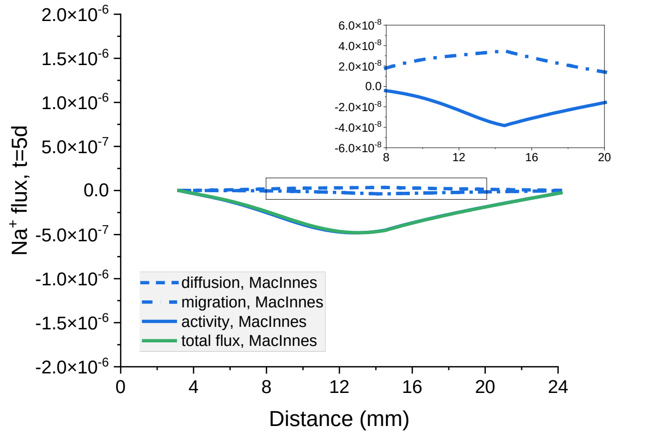
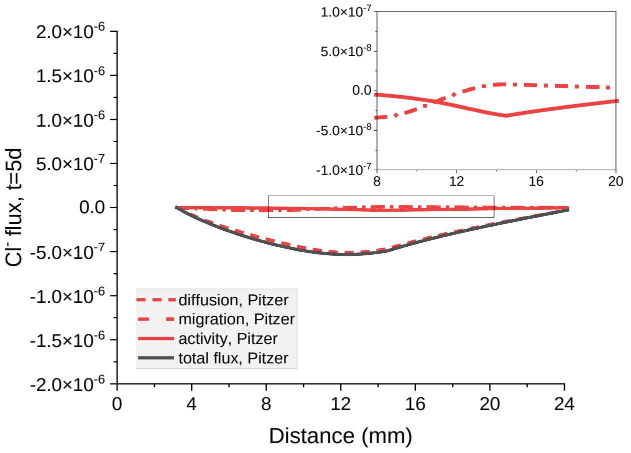	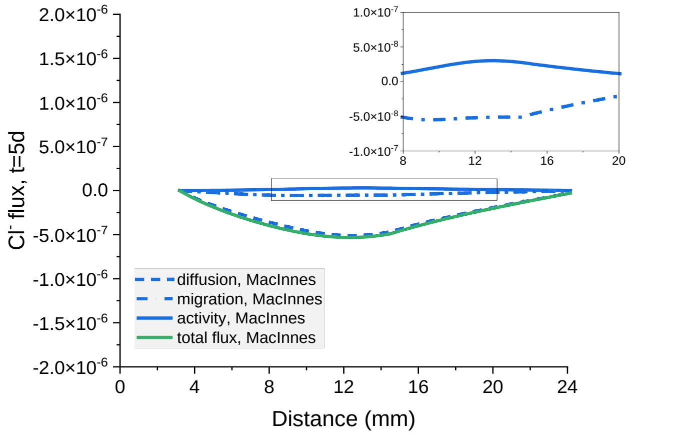
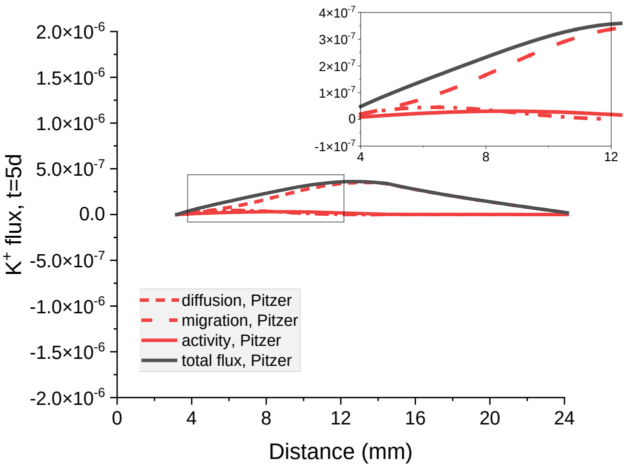	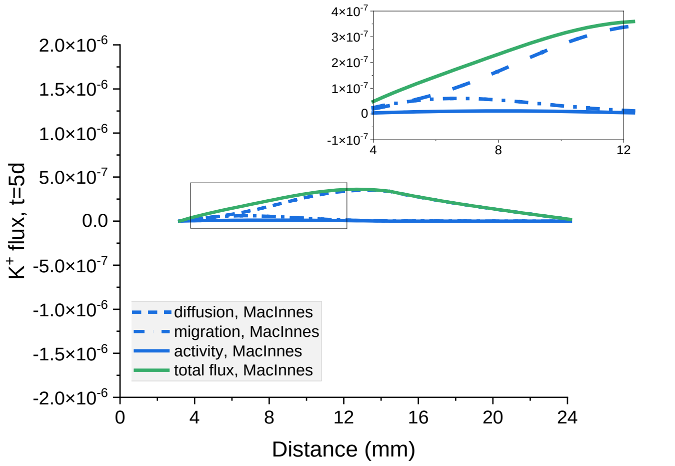

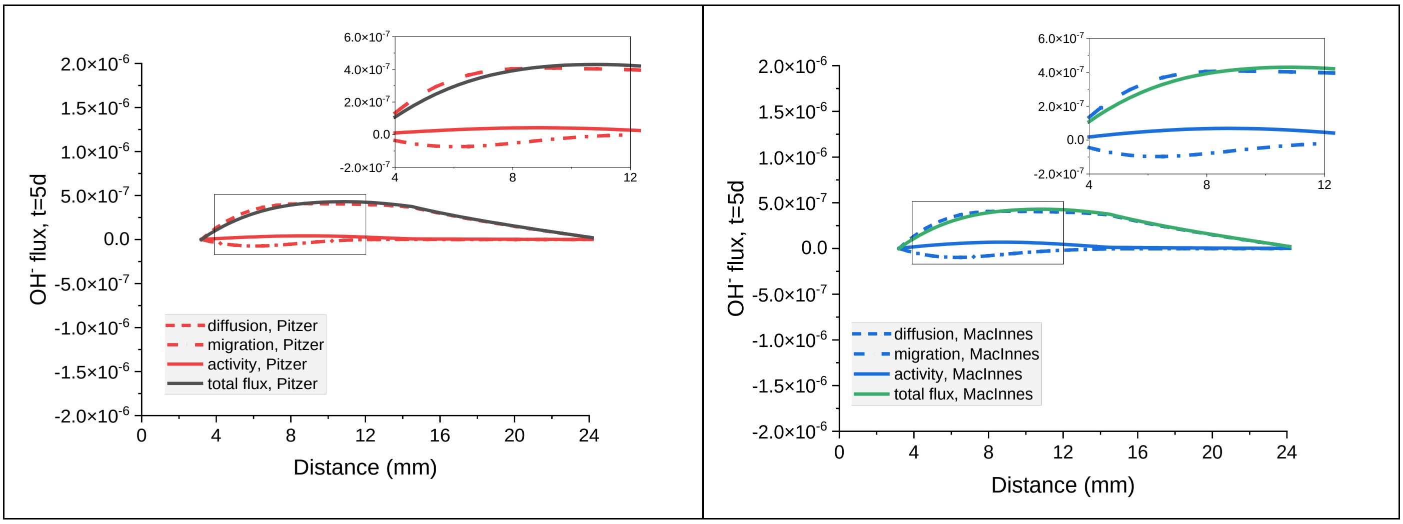

Figure S2. Influence of diffusion, migration and activity terms on the total flux of Na^+, Cl^-, K^+ and OH^- ions in the mortar after 5 days immersion in 5 wt% NaCl water solution for Pitzer a) and MacInnes scaling b).

Supplementary Material 3

Ions concentrations and potential distribution in concrete and mortar samples for dilute solution - comparison

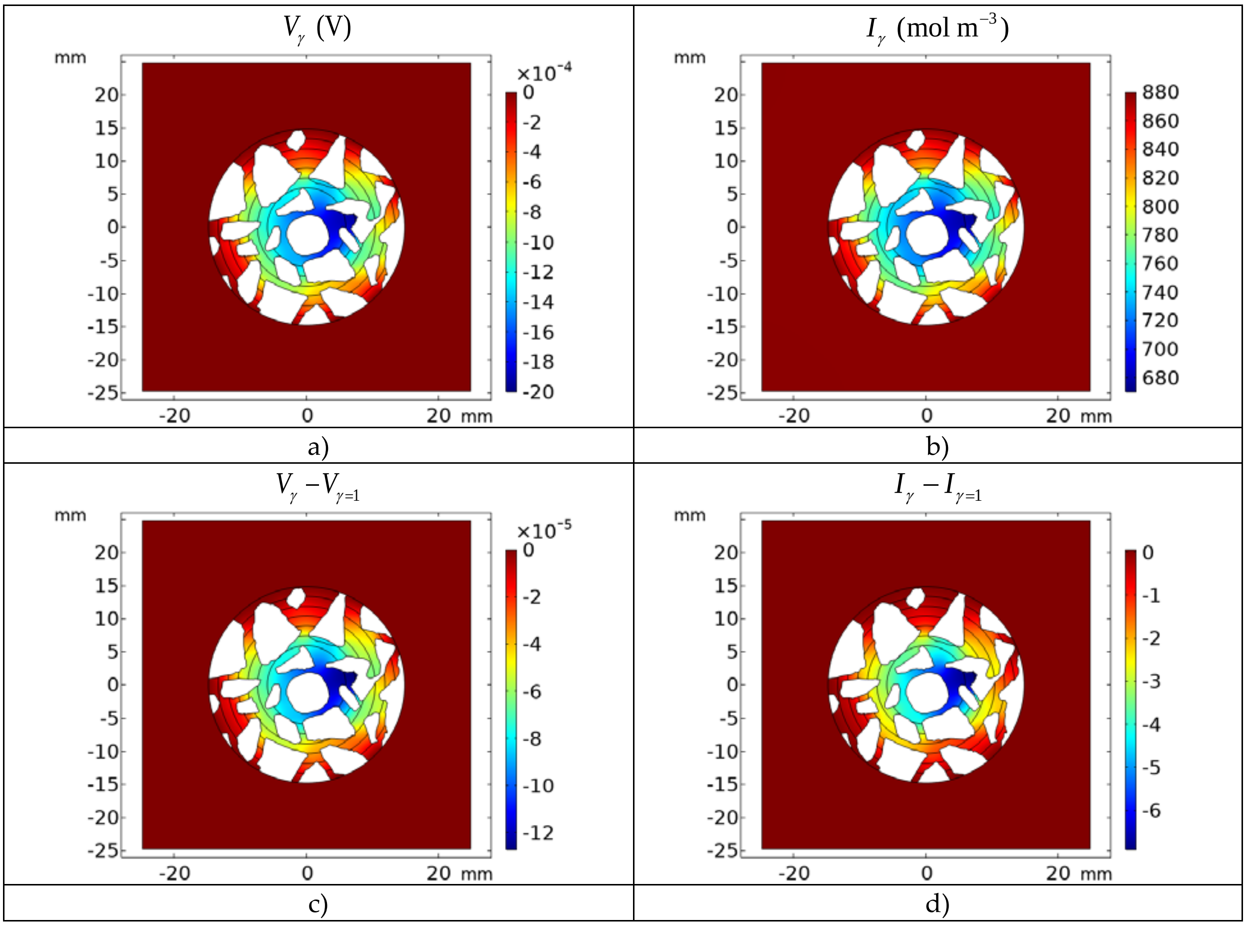

Figure S3. Calculated potential distribution V_γ a) and ionic strength I_γ b) in the cement sample after 1200 h of immersion in 20% NaCl water solution assuming Pitzer activity model. Deviation of Pitzer activity model solution with ideal solution approximation (γ=1) of potential V_γ- $V_{\gamma=1}$ c); and ionic strength I_γ- $I_{\gamma=1}$ d).

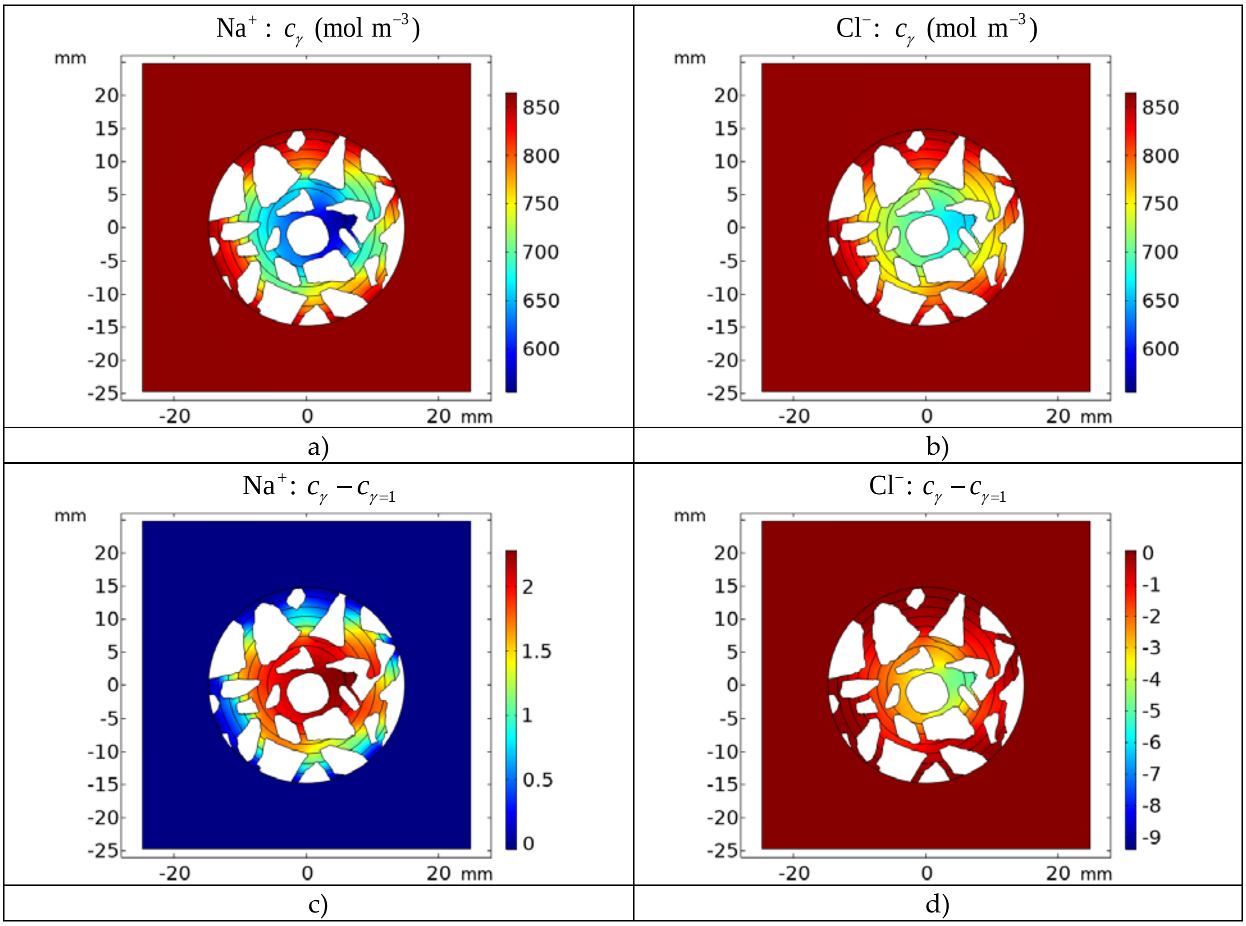

Figure S4. Calculated concentration distribution c_γ of Na^+ a) and Cl^- b) in the cement sample after 1200 h of immersion in 20% NaCl water solution assuming Pitzer activity model. Deviation of Pitzer activity model solution with ideal solution approximation (γ=1) c_γ- $c_{\gamma=1}$ for Na^+ c) and Cl^- d) ions.

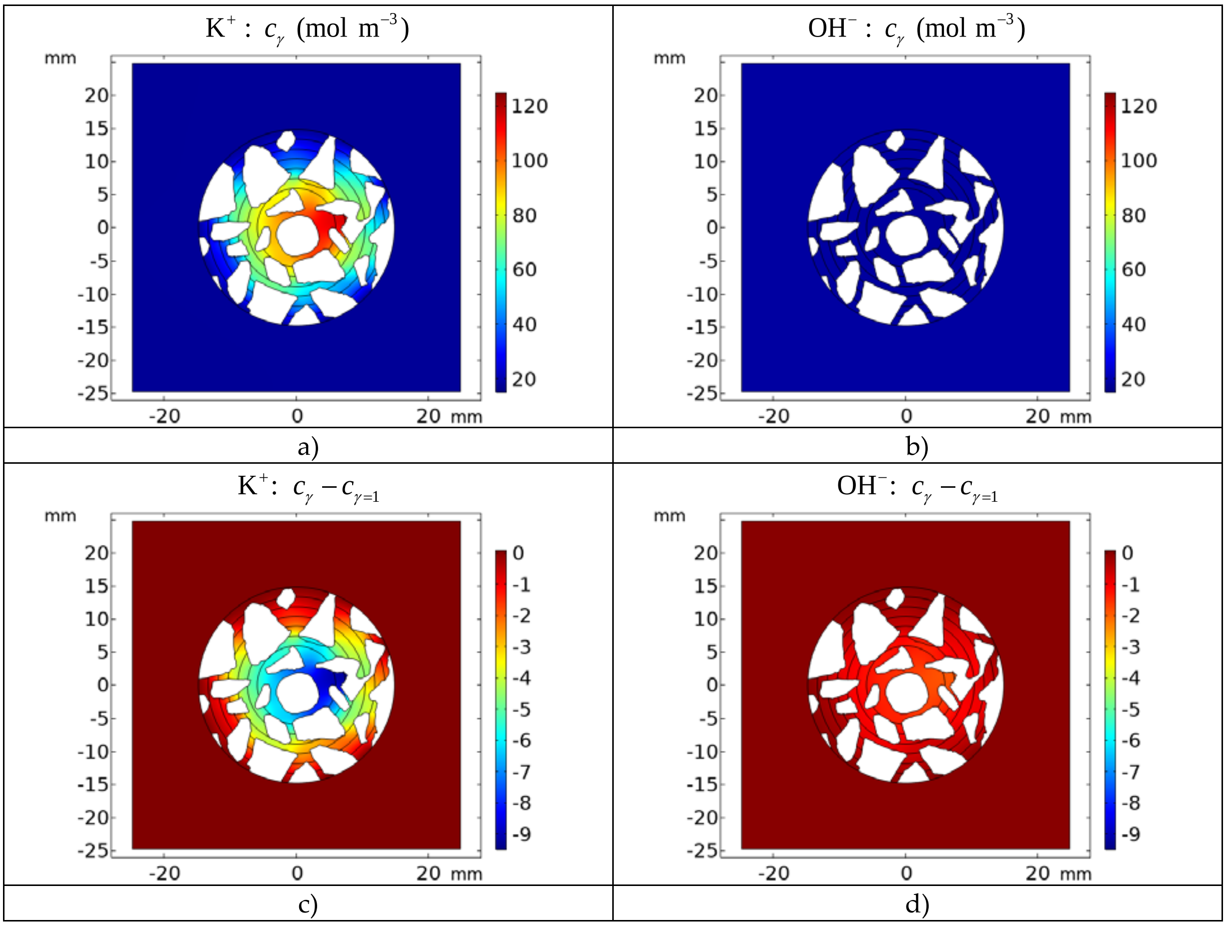

Figure S5. Calculated concentration distribution c_γ of K^+ a) and OH^- b) in the cement sample after 1200 h of immersion in 20% NaCl water solution assuming Pitzer activity model. Deviation of Pitzer activity model solution with ideal solution approximation (γ=1) c_γ- $c_{\gamma=1}$ for K^+ c) and OH^- d) ions.

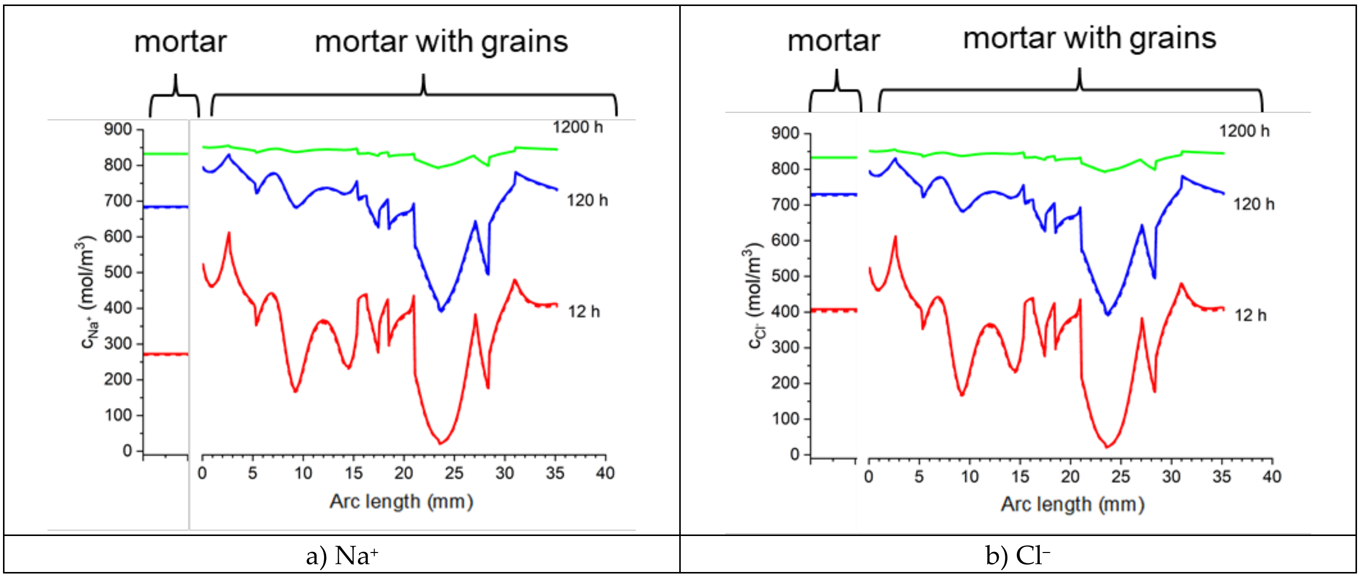

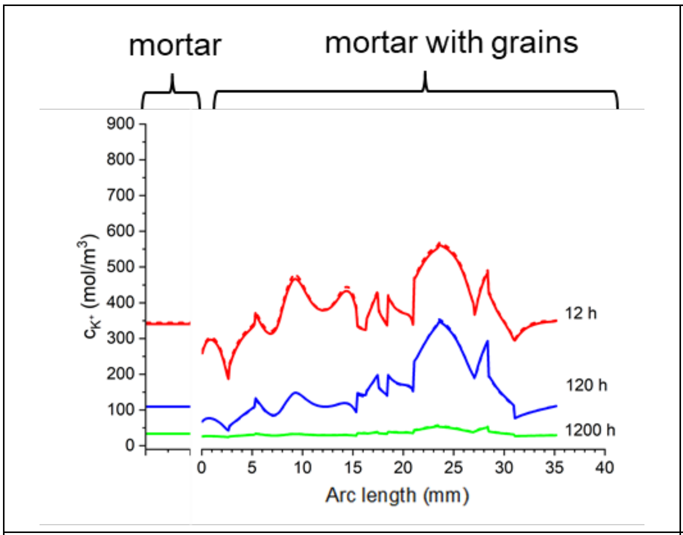

c) K^+

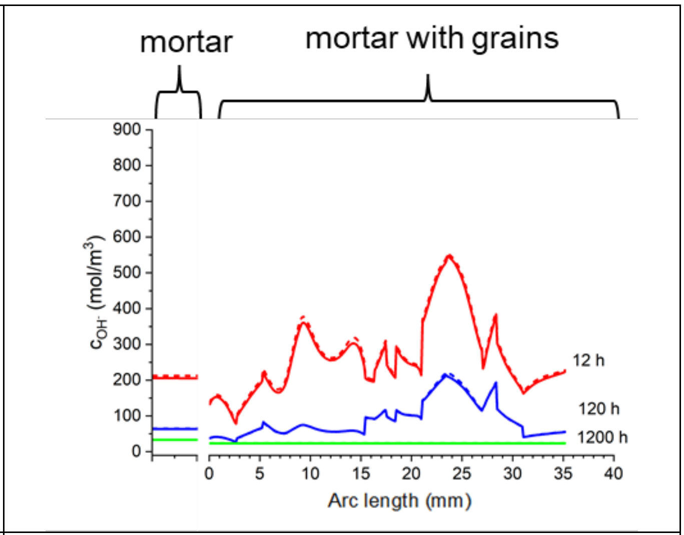

d) OH^-

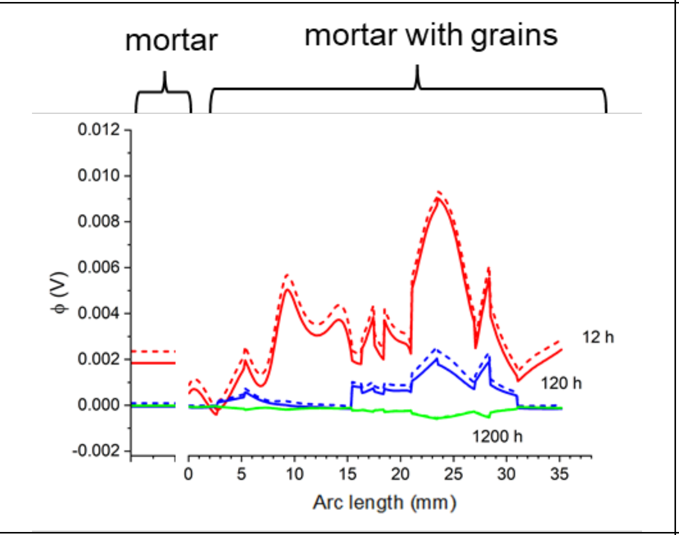

e) potential

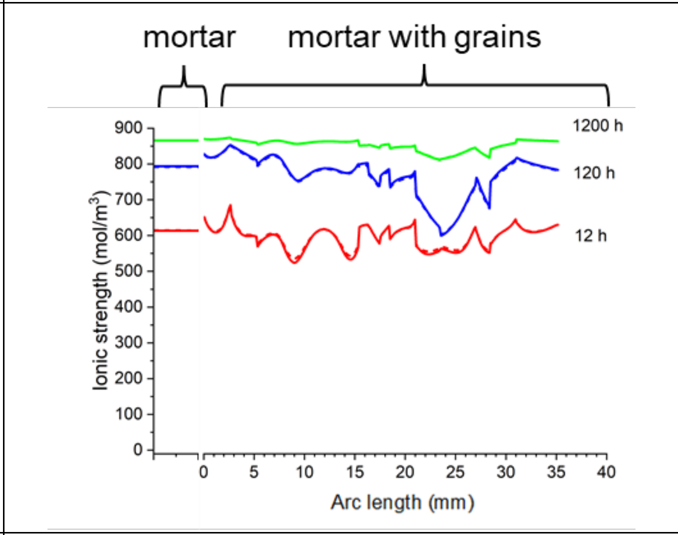

f) ionic strength

Figure S6. Calculated: ions concentrations: a) Na^+, b) Cl^- , c) K^+, d) OH^- e) potential (ϕ) and f) ionic strength in mortar and concrete samples at the distance 13 mm from the centre of the sample in the sample in 5 % NaCl water solution (along a blue line). Solid lines correspond to solution for Pitzer activity model and dashed line neglecting activities ($\gamma = 1$).

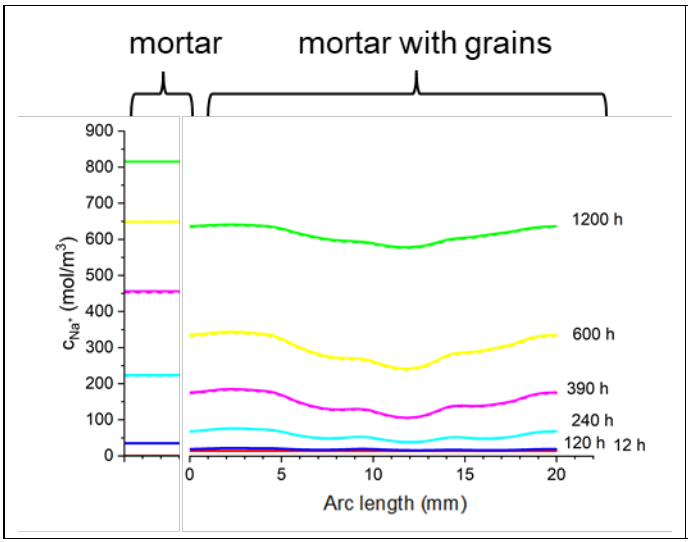

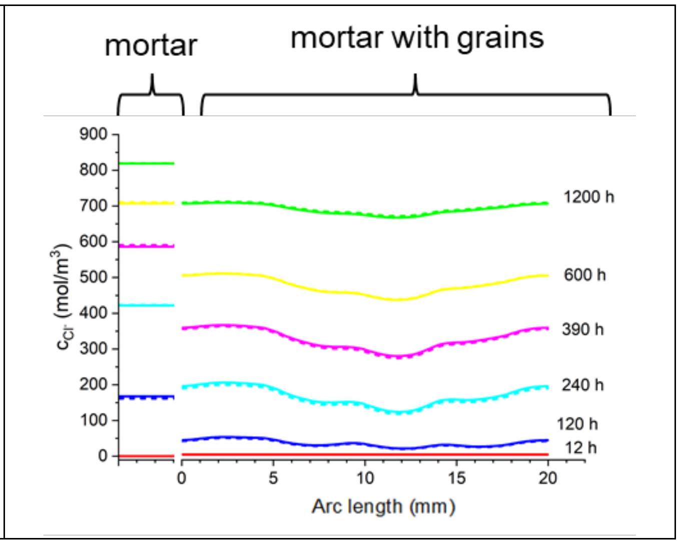

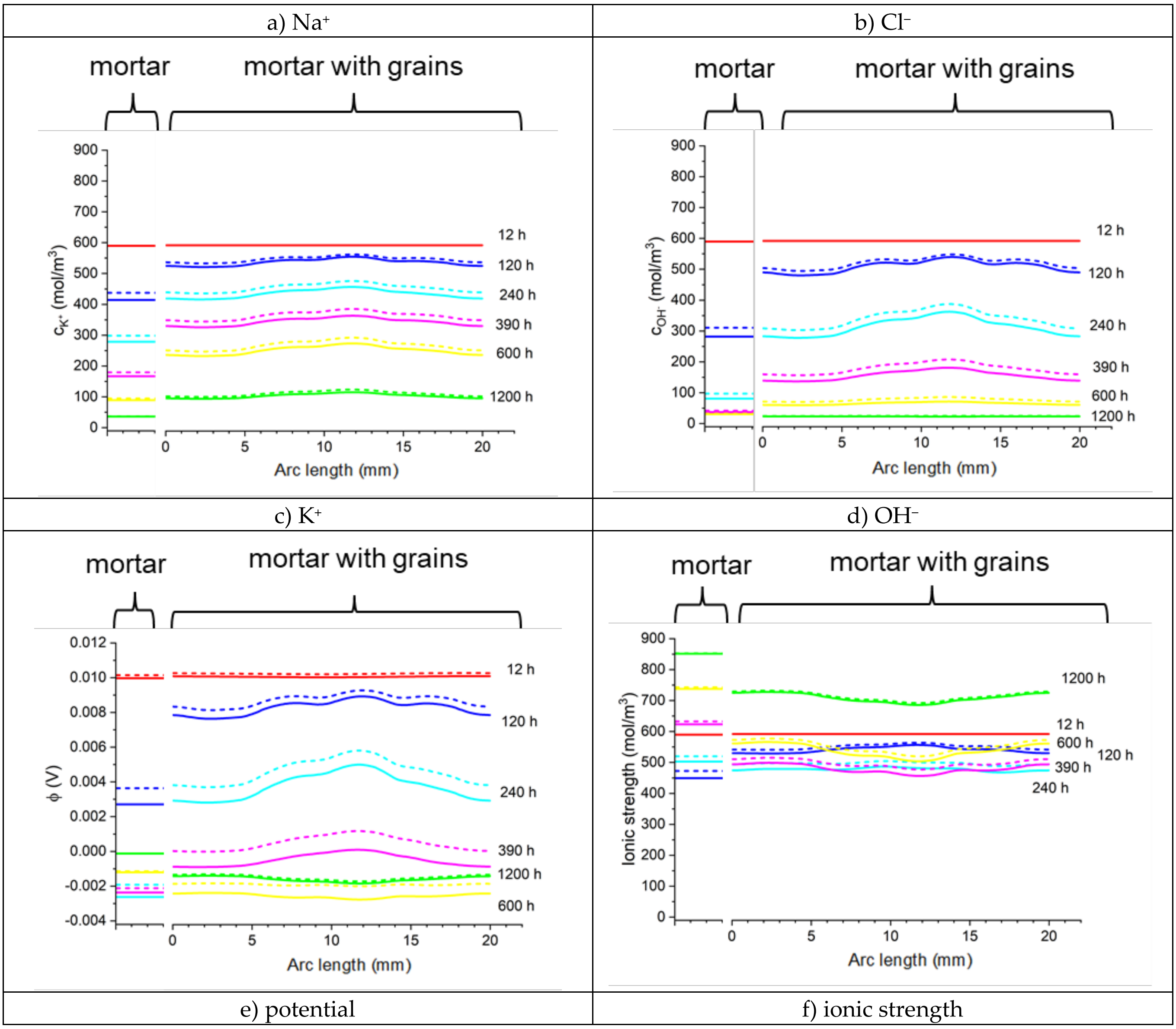

Figure S7. Calculated: ions concentrations: a) Na^{+}, b) Cl^{-} , c) K^{+}, d) OH^{-}, e) potential (ϕ) and f) ionic strength in mortar and concrete samples at the surface of the rod in 5 % NaCl water solution (along a blue line). Solid lines correspond to solution for Pitzer activity model and dashed line neglecting activities ($\gamma = 1$).
